# KDM4A Erases the H3R17me2a Mark, Facilitating Chromosome Condensation

**DOI:** 10.1002/advs.202514281

**Published:** 2026-02-26

**Authors:** Yena Cho, Jee Won Hwang, Gyu Hwan Hyun, Sangkyu Lee, Dae‐Geun Song, Su‐Nam Kim, Yong Kee Kim

**Affiliations:** ^1^ Muscle Physiome Research Center and Research Institute of Pharmaceutical Sciences Sookmyung Women's University Seoul Republic of Korea; ^2^ College of Pharmacy Sookmyung Women's University Seoul Republic of Korea; ^3^ School of Pharmacy Sungkyunkwan University Suwon Republic of Korea; ^4^ Natural Products Research Institute KIST Gangneung Gangneung Republic of Korea; ^5^ Natural Product Applied Science KIST School University of Science and Technology Gangneung Republic of Korea

**Keywords:** CARM1, demethylation, KDM4A, methylation, mitosis, phosphorylation

## Abstract

Chromosome condensation during mitosis is essential for proper cell division. Histone modifications, particularly the dynamic regulation of active and repressive marks, play a crucial role in this process. However, the mechanisms controlling the temporal interplay between these marks remain unclear. This study revealed the reversible regulation of the active mark H3R17me2a and its interplay with the repressive mark H3K9me3. Early in mitosis, CARM1, which is responsible for H3R17me2a formation, is inactivated, while KDM4A gains arginine demethylase activity to remove H3R17me2a via PKCα‐mediated phosphorylation. This demethylation step allows Suv39h1 to facilitate H3K9me3 accumulation, promoting the recruitment of the chromosomal passenger complex and subsequent H3S10 phosphorylation, thus completing chromosome condensation. These findings provide novel insights into the interplay between histone modifications during mitosis and the precise mechanisms governing chromosome dynamics.

## Graphical abstract

This study reveals a reversible histone modification switch governing chromosome condensation during mitosis. PKCα‐activated KDM4A removes H3R17me2a, permitting Suv39h1‐driven H3K9me3 deposition. This epigenetic transition recruits the chromosomal passenger complex and triggers Aurora B‐dependent H3S10 phosphorylation, coordinating chromatin remodeling required for faithful mitotic chromosome condensation.

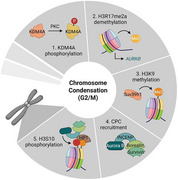

## 1 Introduction

Chromosome condensation is critical for accurate chromosome segregation during mitosis [1]. This process is driven by dynamic histone modifications, such as phosphorylation and methylation [2, 3]. Among the well‐characterized mitotic‐specific modifications, H3S10 phosphorylation (H3S10ph) and H3K9 trimethylation (H3K9me3) play key roles [4, 5]. H3K9me3 acts as a binding platform for heterochromatin protein 1 (HP1) [6, 7], which coordinates the localization and activation of the chromosomal passenger complex (CPC) [8, 9]. CPC, composed of Aurora B kinase, INCENP, borealin, and survivin, ensures chromosomal stability during mitosis [10]. The early recruitment of CPC by HP1 activates Aurora B, promoting H3S10ph, and driving chromatin compaction [9]. Although these modifications have been well‐studied, the interplay between other histone modifications, such as arginine methylation, and chromosome condensation has been less explored. A recent study has shown that the asymmetric dimethylation of H3 at arginine 2 (H3R2me2a) by PRMT6 also recruits CPC to the chromosome arms, contributing to H3S10ph establishment [11]. This suggests a broader crosstalk between methylation and phosphorylation during mitosis. However, the roles of other histone modifications in this process remain unclear.

Coactivator‐associated arginine methyltransferase 1 (CARM1, also known as PRMT4) has emerged as a key regulator of the cell cycle [12, 13, 14]. The phosphorylation of CARM1 at S217 and S229 during the G2/M transition inhibits its enzymatic activity and triggers its translocation from the nucleus to the cytoplasm [15, 16]. In the nucleus, CARM1 regulates the proteolytic degradation of cyclin‐dependent kinase 1 (CDK1); thus, its cytoplasmic translocation stabilizes nuclear CDK1, promoting mitotic entry through the phosphorylation of its substrates [17]. However, whether the enzymatic inhibition of CARM1 is necessary during mitosis is not fully understood. Chromosome condensation, a transcriptionally silent process, requires the loss of active marks (H3K9ac [18] and H3K14ac [18]) and the accumulation of repressive marks (H3R2me2a [11] and H3K9me3 [19, 20]). Given that CARM1 generates transcriptionally active marks such as H3R17me2a and H3R26me2a [21, 22, 23, 24], we hypothesized that CARM1 inactivation is critical for proper chromosome condensation by preventing the formation of these active marks. In this study, we investigated whether CARM1 inactivation is essential for mitotic progression and how H3R17me2a is dynamically regulated during mitosis.

## 2 Results

### 2.1 H3R17me2a Reversibly Decreases to Facilitate Chromosome Condensation During Mitosis

**FIGURE 1:**
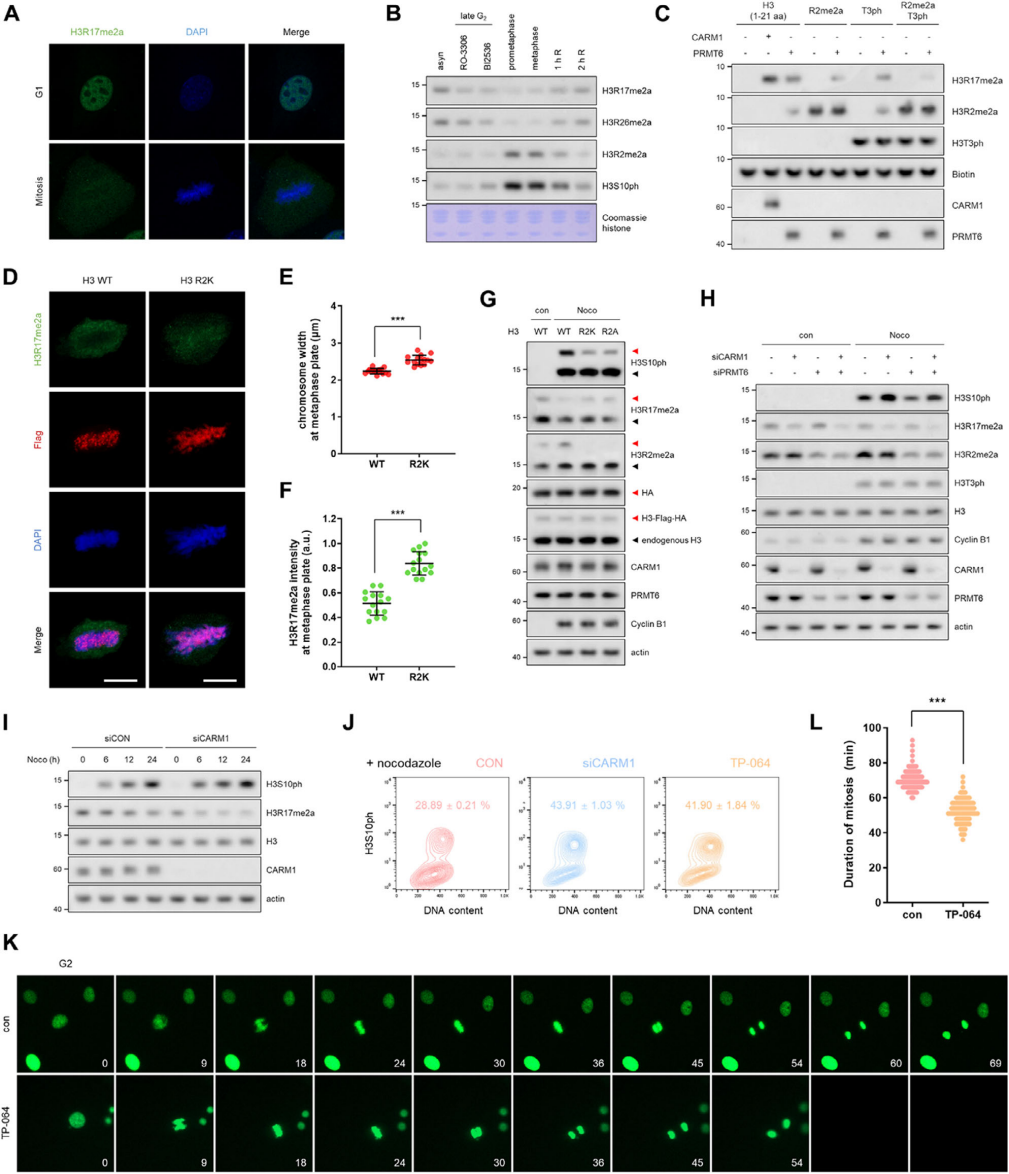
H3R17me2a reversibly decreases to facilitate chromosome condensation during mitosis. (A), Confocal images of H3R17me2a (green) and DAPI (blue) in 10T1/2 cells at interphase and metaphase. (B), Western blots of histones from cells arrested in late G2 (RO‐3306 or BI2536), prometaphase (nocodazole), or metaphase (nocodazole release with MG132). (C), In vitro methylation assay using biotinylated histone H3 peptides (1–21 aa; AR^*^T^*^KQTARKSTGGKAPRKQLA‐GGK(biotin)) and beads‐captured PRMT. (D–F), Confocal images of H3R17me2a (green), Flag (red), and DAPI (blue) in cells overexpressing H3‐Flag‐HA‐WT or ‐R2K (D). Quantification of the chromosome width (E) and H3R17me2a intensity (F) in the metaphase plate of Flag‐positive cells. Scale bars, 5 µm. (G), Western blots of lysates from cells overexpressing H3‐Flag‐HA‐WT, ‐R2K, or ‐R2A. Red arrowheads indicate Flag‐HA‐tagged H3, whereas black arrowheads indicate endogenous H3. (H), Western blots of lysates from cells treated with nocodazole after the knockdown of CARM1 and/or PRMT6. (I), Western blots of lysates from cells treated with nocodazole for 6, 12, or 24 h after CARM1 knockdown. (J), Levels of H3S10ph measured by FACS in cells treated with CARM1 siRNA or inhibitor for 72 h. Data are presented as the mean ± SD (n = 3). (K,L), After treatment with the CARM1 inhibitor for 72 h, 10T1/2 cells stably expressing GFP‐H2B were filmed for 5 h. Representative images are shown (K), and the duration of mitosis was determined (L).

To investigate the dynamic changes in histone substrate methylation by CARM1 during mitosis, we analyzed the arginine methylation of histone proteins throughout the cell cycle in 10T1/2 cells, normal mouse embryonic fibroblasts. As expected, the levels of the CARM1‐dependent marks H3R17me2a and H3R26me2a began to decrease from the G2 phase, coinciding with the inhibition of CARM1 enzymatic activity at mitotic entry, and recovered after mitotic exit (Figure 1A,B; Figure S1A–E). In contrast, H3R2me2a, which is catalyzed by PRMT6, increased during mitosis (Figure 1B). Since PRMT6 has been reported to catalyze H3R17 methylation, albeit less efficiently than CARM1 [25], it is possible that PRMT6 could partially compensate for the decrease in H3R17me2a during mitosis. To address this potential confounding factor, we performed in vitro methylation assays using histone peptide, H3R2me2aT3ph, designed to mimic the mitotic histone context. PRMT6 could catalyze H3R17me2a in the unmodified H3 peptide but is much less efficient when H3R2me2aT3ph modifications are present (Figure 1C), arguing against a significant compensatory contribution of PRMT6 to H3R17me2a generation during mitosis. In a previous study, we demonstrated that H3R2me2a was essential for chromosome condensation, as shown by the increased metaphase plate width in cells overexpressing H3R2 methylation‐deficient mutants (H3R2K or H3R2A) [11] (Figure 1D,E). Interestingly, the reduction in H3R17me2a levels during mitosis in these cells was less pronounced than that in wild‐type (WT) cells (Figure 1D–G). This observation is consistent with the notion that PRMT6‐mediated R17 methylation preferentially occurs when R2 remains unmethylated, in agreement with our in vitro assays (Figure 1C). Thus, we hypothesized that both H3R17me2a and H3R2me2a affect H3S10ph and chromosome condensation. To specifically assess mitosis‐associated chromatin states, cells were arrested in mitosis using nocodazole. In nocodazole‐arrested mitotic cells, CARM1 depletion led to a greater reduction in H3R17me2a levels and a concurrent increase in H3S10ph levels than the corresponding controls (Figure 1H). In contrast, PRMT6 knockdown, which reduced H3R2me2a levels, did not significantly affect H3R17me2a levels or lead to a substantial increase in H3S10ph levels (Figure 1H; Figure S1F), indicating that H3R17 plays a critical role in H3S10ph. Indeed, the reduction in H3R17me2a levels caused by CARM1 knockdown or specific inhibitor (TP‐064) increased nocodazole‐induced H3S10ph levels (Figure 1I,J; Figure S1G,H) and shortened mitotic duration (Figure 1K,L; Video S1). These findings suggest that the inhibition of CARM1 during mitosis reduces H3R17me2a, which in turn triggers an increase in H3S10ph, facilitating chromosome condensation.

### 2.2 Reduction in H3R17me2a Increases Aurora B Levels and Chromatin Binding, Inducing H3S10ph

**FIGURE 2:**
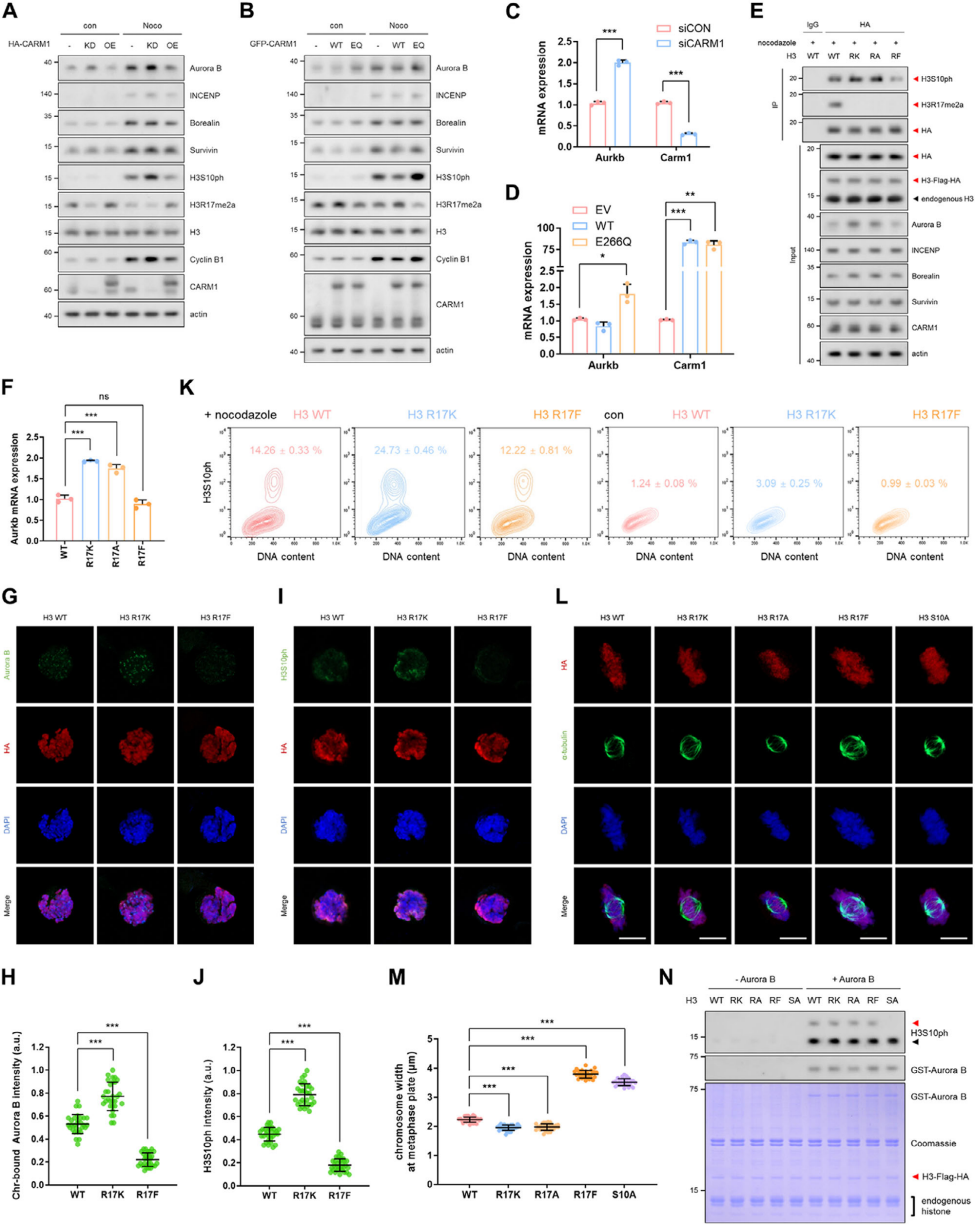
Reduction in H3R17me2a increases Aurora B levels and chromatin binding, inducing H3S10ph. (A), Western blots of lysates from 10T1/2 cells with CARM1 knockdown or overexpression. (B), Western blots of lysates from cells incubated with CARM1‐WT or ‐E266Q for 48 h. (C,D), mRNA levels of *Aurkb* and *Carm1* in cells transfected with CARM1 siRNA for 72 h (C) or plasmid for 48 h (D). (E,F), Immunoprecipitation using an anti‐HA antibody (E) and measurement of *Aurkb* mRNA levels (F) in cells overexpressing H3‐Flag‐HA‐WT, ‐R17K, ‐R17A, or ‐R17F. Red arrowheads indicate Flag‐HA‐tagged H3, whereas black arrowheads indicate endogenous H3. (G–J), Representative immunofluorescence images and quantitative intensity analyses of Aurora B (G,H) and H3S10ph (I,J) in cells overexpressing H3‐Flag‐HA‐WT, ‐R17K, or ‐R17F. (K), Levels of H3S10ph measured by FACS in cells overexpressing H3‐Flag‐HA‐WT, ‐R17K, or ‐R17F. Data are presented as the mean ± SD (n = 3). (L,M), Confocal images of HA (red), α‐tubulin (green), and DAPI (blue) in cells overexpressing H3‐Flag‐HA‐WT, ‐R17K, ‐R17A, ‐R17F, or ‐S10A. Scale bars, 5 µm (L). Quantification of metaphase plate chromosome width in HA‐positive cells (M). Cells were synchronized at metaphase by treatment with nocodazole and MG132. Chromosome width was determined by measuring spindle width, defined as the distance between the two outermost edges of the α‐tubulin signal perpendicular to the spindle axis. Measurements were based on the spatial extent of the signal rather than fluorescence intensity. (N), In vitro kinase assay using histone extracts from cells overexpressing H3‐Flag‐HA (WT, R17K, R17A, R17F, or S10A) and recombinant Aurora B protein.

To investigate how decreased H3R17me2a levels influence the increase in H3S10ph levels, we first examined the levels of CPC subunits in cells where CARM1 was either knocked down or overexpressed (WT or E266Q mutant). We observed that only Aurora B levels decreased with CARM1 WT overexpression and increased with CARM1 knockdown or E266Q overexpression at both protein and mRNA levels (Figure 2A–D). This suggests that despite H3R17me2a being a well‐established active mark [23, 24], it may repress *Aurkb* transcription, thereby regulating Aurora B abundance. To confirm this further, we designed experiments using ΔNLS‐CARM1 (Δ348–381) and CARM1 inhibitor (Figure S2A–D). TP‐064 treatment, which reduced H3R17me2a levels [26], increased Aurora B protein and mRNA levels (Figure S2A,B). Furthermore, the restoration of H3R17me2a in CARM1 knock‐out (KO) cells via rescue with WT CARM1, but not ΔNLS‐CARM1, led to a reduction in Aurora B protein and mRNA levels (Figure S2C,D). Direct evidence was provided by overexpressing the H3R17K or H3R17A form, which increased both Aurora B and H3S10ph levels (Figure 2E–J). This resulted in faster nocodazole‐induced mitotic arrest and a narrower metaphase plate width compared to overexpressing the WT (Figure 2K–M). In contrast, overexpression of the methylation‐mimetic H3R17F or phosphorylation‐deficient H3S10A led to the opposite results (Figure 2E–M; Figure S2E,F), supporting the idea that decreased H3R17me2a upregulates *Aurkb* transcription, promoting H3S10ph, chromosome condensation, and mitotic entry. Notably, Aurora B kinase activity toward its substrates was not altered by the H3R17 methylation status, as Aurora B efficiently phosphorylated its substrates regardless of R17 methylation (Figure 2N; Figure S2G). Collectively, these findings suggest that during mitosis, CARM1 suppression leads to a reduction in H3R17me2a, which in turn increases both Aurora B expression and its chromatin binding, ultimately elevating H3S10ph levels and promoting chromosome condensation and mitotic progression (Figure S2H–J).

### 2.3 Reduction in H3R17me2a During Mitosis Enhances the Binding of Suv39h1 to Chromatin, Leading to an Increase in H3K9me3

**FIGURE 3:**
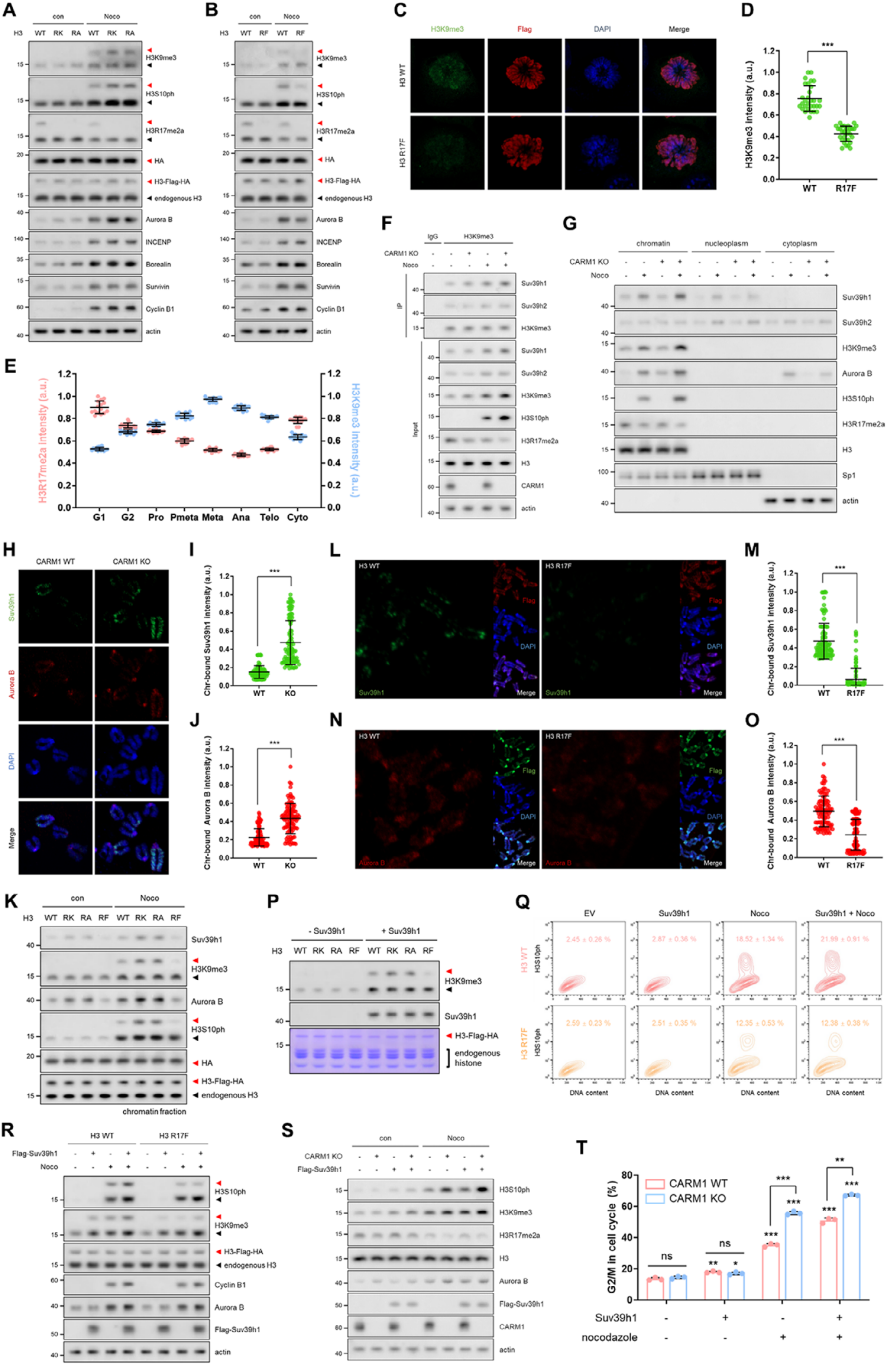
Reduction in H3R17me2a during mitosis enhances the binding of Suv39h1 to chromatin, leading to an increase in H3K9me3. (A,B), Western blots of lysates from 10T1/2 cells overexpressing the H3R17 methylation‐deficient mutant (A) and the methylation‐mimetic mutant (B). Red arrowheads indicate Flag‐HA‐tagged H3, whereas black arrowheads indicate endogenous H3. (C,D), Confocal images of H3K9me3 (green), Flag (red), and DAPI (blue) in cells overexpressing H3‐Flag‐HA‐WT or ‐R17F (C). H3K9me3 intensity in Flag‐positive cells was quantified (D). (E), The intensities of H3R17me2a and H3K9me3 throughout the cell cycle were analyzed via immunostaining. Quantification of representative images shown in Figure S3A. (F,G), Immunoprecipitation using an anti‐H3K9me3 antibody (F) and chromatin fractionations (G) in CARM1‐WT or ‐KO cells treated with nocodazole. (H,I,J), Confocal images of chromosome spreads from nocodazole‐arrested CARM1‐WT or CARM1‐KO cells. Suv39h1 (green), Aurora B (red), and DAPI (blue) are shown (H). Quantification of the fluorescence intensities of chromosome‐bound Suv39h1 (I) and Aurora B (J). (K), Western blots of chromatin fractions from cells overexpressing H3‐Flag‐HA‐WT, ‐R17K, ‐R17A, or ‐R17F. Red arrowheads indicate Flag‐HA‐tagged H3, whereas black arrowheads indicate endogenous H3. (L–O), Representative images and quantification of chromosome‐bound Suv39h1 (green; L,M) and Aurora B (red; N,O) obtained by chromosome‐spreading assays in nocodazole‐arrested cells overexpressing H3‐Flag‐HA‐WT or ‐R17F. (P), In vitro methylation assay using histone extracts from cells overexpressing H3‐Flag‐HA (WT, R17K, R17A, or R17F) and beads‐captured Suv39h1. Red arrowheads indicate Flag‐HA‐tagged H3, whereas black arrowheads indicate endogenous H3. (Q,R), Levels of H3S10ph measured by FACS (Q) and western blots (R) in H3‐Flag‐HA‐WT or ‐R17F overexpressing cells under the indicated conditions. Red arrowheads indicate Flag‐HA‐tagged H3, whereas black arrowheads indicate endogenous H3. (S,T), Western blots (S) and cell cycle profiles analyzed using FACS (T) in CARM1‐WT or ‐KO cells under the indicated conditions.

Next, we sought to determine how the reduction in H3R17me2a increased Aurora B binding to chromosomes. Considering that CPC recruitment to chromosomes depends on the interaction between H3K9me3‐bound HP1 and INCENP [8], we first focused on the crosstalk between H3R17me2a and H3K9me3. Interestingly, H3K9me3 levels were increased in H3R17 methylation‐deficient mutants (H3R17K and H3R17A), whereas H3K9me3 levels were reduced in the H3R17 methylation‐mimetic mutant (H3R17F) (Figure 3A–D). This pattern was consistent with the changes in Aurora B expression and H3S10ph levels we observed (Figure 3A,B). Overall, similar to the relationship between H3R17me2a and H3S10ph, changes in the H3R17me2a and H3K9me3 marks throughout the cell cycle showed an inverse correlation (Figure 3E; Figure S3A). Even when H3R17me2a levels were reduced by CARM1 knockdown, H3K9me3 levels increased (Figure S3B–D), strongly suggesting that H3R17 methylation plays a role in regulating H3K9 methylation. Since Suv39h1 and Suv39h2 are well‐known enzymes that catalyze H3K9me3 [27, 28], we investigated whether H3R17me2a affects their binding to chromatin. Under conditions where H3R17me2a levels were reduced (CARM1 KO, knockdown, or inhibition), Suv39h2 was unaffected; however, Suv39h1 bound more strongly to chromatin, increasing H3K9me3 levels, which in turn led to the increased binding of Aurora B to chromatin and elevated H3S10ph levels (Figure 3F–J; Figure S3E–L). Only in the group overexpressing H3R17F was Suv39h1 recruitment suppressed, which was followed by a decrease in H3K9me3 and the levels of chromatin‐bound Aurora B and H3S10ph (Figure 3K–O). These findings suggest that the interaction between H3R17me2a and H3K9me3 is mediated by the binding of Suv39h1 to chromatin. Furthermore, Suv39h1 preferentially catalyzed K9me3 on H3, which was unmethylated at R17, whereas Suv39h2 induced K9me3 regardless of R17 methylation (Figure 3P; Figure S3M). This result demonstrates that H3R17 methylation alters the substrate preference of Suv39h1. Collectively, these observations strongly suggest that H3R17me2a acts as a key mark that suppresses chromosome condensation and mitotic entry through Suv39h1‐mediated H3K9me3 and subsequent H3S10ph. Indeed, H3R17F weakened nocodazole‐induced mitotic arrest and suppressed the increase in H3K9me3 and H3S10ph levels in cells overexpressing Suv39h1 (Figure 3Q,R). In contrast, Suv39h1 overexpression in CARM1 KO cells increased H3K9me3 and H3S10ph levels, thereby enhancing nocodazole‐induced mitotic arrest (Figure 3S,T; Figure S3N).

### 2.4 KDM3A and KDM4A Possess Arginine Demethylase (RDM) Activity That Erases the H3R17me2a Mark In Vivo

**FIGURE 4:**
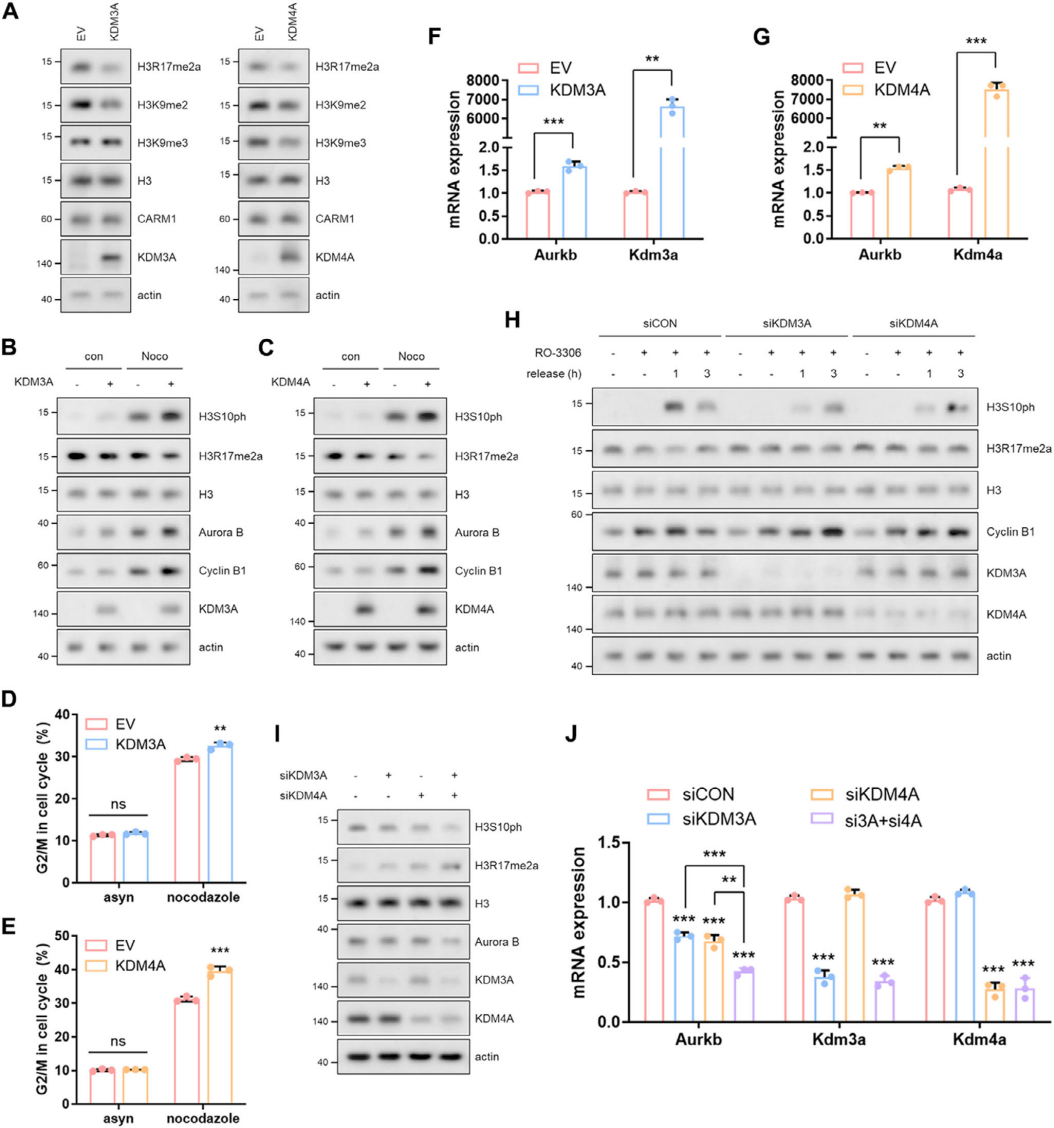
KDM3A and KDM4A possess RDM activity that erases the H3R17me2a mark in vivo*. (*A), Western blots of lysates from 10T1/2 cells incubated with KDM3A or KDM4A for 48 h. (B–E), Western blots (B,C) and cell cycle profiles analyzed using FACS (D,E) in nocodazole‐treated cells after transfection with KDM3A or KDM4A. (F,G), mRNA levels of *Aurkb* and *Kdm* in cells incubated with KDM3A (F) or KDM4A (G) for 48 h. (H), Western blots of lysates from cells arrested at the G2 phase and then released after knocking down KDM3A and/or KDM4A. (I,J), Western blots (I) and mRNA levels (J) in cells with knocked down KDM3A and/or KDM4A.

While we have elucidated how the reduction of H3R17me2a contributes to chromosome condensation, the fundamental question of how H3R17me2a levels are dynamically regulated during mitosis remains unanswered. Arginine methylation is considered a reversible process in vivo; however, bona fide arginine demethylases (RDMs) have not yet been identified. A recent study showed that certain lysine demethylases (KDMs), including KDM3A and KDM4A, exhibit RDM activity, albeit mainly in vitro [29]. This suggests that arginine demethylation by some KDMs occurs in specific cellular contexts in vivo [30, 31, 32]. We then examined whether KDM3A, KDM4A, KDM4C, KDM4E, KDM5C, KDM6B, KDM7B, or Jumonji domain‐containing protein 6 (JMJD6) could demethylate H3R17me2a in vivo. Among these, both KDM3A and KDM4A displayed robust RDM activity, representing a functional expansion beyond their well‐established KDM activity (Figure 4A; Figure S4A,B). Therefore, we focused our investigation on these two enzymes. The overexpression of KDM3A or KDM4A led to a dramatic reduction in H3R17me2a levels, accompanied by increased *Aurkb* transcription, elevated H3S10ph levels in response to nocodazole treatment, and pronounced mitotic arrest (Figure 4B–G; Figure S4C,D). These results closely mirrored the effects observed in CARM1‐deficient cells (Figure 1I,J,2C). Conversely, the knockdown of KDM3A or KDM4A impaired the reduction of H3R17me2a during mitotic progression following RO‐3306 release (Figure 4H). This resulted in a slower increase in H3S10ph levels compared to the controls, indicating a marked delay in mitotic progression (Figure 4H). Collectively, these findings suggest that KDM3A and KDM4A serve as counterparts of CARM1, regulating H3R17me2a levels and then influencing both Aurora B levels and the G2/M transition (Figure 4I,J; Figure S4E–H). Additionally, we found that the overexpression or knockdown of KDM4C, which did not erase H3R17me2a (Figure S4B) although it is most homologous to KDM4A [33], did not affect the levels of H3S10ph and Aurora B (Figure S4I,J), clarifying the contribution of H3R17 to mitotic entry.

### 2.5 KDM4A is Phosphorylated by PKCα During Mitosis, Acquiring H3R17me2a Demethylation Activity

**FIGURE 5:**
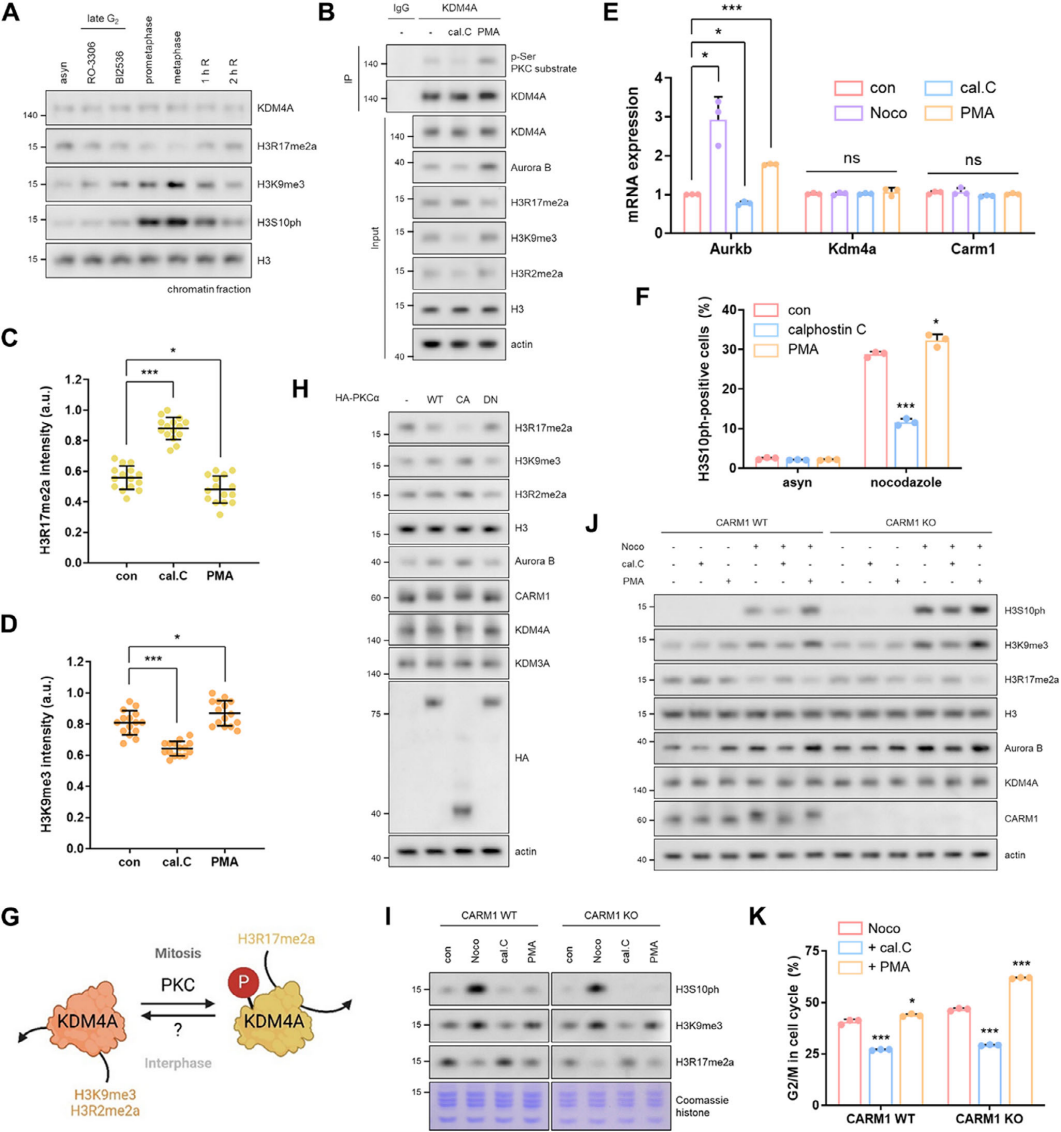
KDM4A is phosphorylated by PKCα during mitosis, acquiring H3R17me2a demethylation activity. (A), Western blots of the chromatin fractions from cells arrested at the G2 (RO‐3306 or BI2536), prometaphase (nocodazole), or metaphase (nocodazole release with MG132). (B–D), Immunoprecipitation using an anti‐KDM4A antibody (B) and intensities of H3R17me2a (C) and H3K9me3 as determined via immunostaining (D) in cells treated with 0.5 µm calphostin C or 100 nm PMA for 12 h. (E), mRNA levels of *Aurkb*, *Kdm4a*, and *Carm1* in cells treated with nocodazole, calphostin C, or PMA for 12 h. (F), Levels of H3S10ph measured by FACS in cells added with nocodazole after calphostin C or PMA pretreatment. Data are presented as the mean ± SD (n = 3). (G), Schematic representation of the function of KDM4A depending on PKC‐mediated phosphorylation. (H), Western blots of lysates from cells overexpressing PKCα‐WT, ‐CA, or ‐DN. (I), Western blots of histones from CARM1‐WT or ‐KO cells treated with nocodazole, calphostin C, or PMA for 12 h. (J,K), Western blots (J) and cell cycle profiles analyzed using FACS (K) in CARM1‐WT or ‐KO cells added with nocodazole after calphostin C or PMA pretreatment.

A previous study showed that recombinant KDM3A and KDM4A failed to demethylate the H3R17me2a peptide in vitro [29]. However, our in vivo experiments revealed that KDM3A and KDM4A exhibited RDM activity, effectively erasing the H3R17me2a mark. Based on this, we hypothesized that KDM3A and KDM4A acquire the ability to demethylate H3R17me2a during mitosis. Given that a massive wave of phosphorylation occurs during mitosis [34], we first examined whether KDM3A or KDM4A was phosphorylated at this stage. A band shift in KDM4A was observed during the late G2 phase and mitosis, indicating phosphorylation (Figure S5A). Indeed, KDM4A phosphorylation was significantly increased in nocodazole‐arrested mitotic cells, whereas no changes were detected for KDM3A (Figure S5B,C). Unlike KDM1A, which is phosphorylated and released from chromatin during mitosis [35], KDM4A remained chromatin‐bound regardless of its phosphorylation status (Figure 5A; Figure S5D), suggesting that KDM4A continues to regulate chromatin dynamics throughout mitosis. Using the Scansite database (https://scansite4.mit.edu), we identified protein kinase C (PKC) as the kinase responsible for KDM4A phosphorylation. Supporting this prediction, KDM4A phosphorylation increased upon treatment with the PKC activator (phorbol 12‐myristate 13‐acetate; PMA) and was strongly inhibited by the PKC inhibitor (calphostin C) (Figure 5B). These treatments did not affect the chromatin binding ability of KDM4A (Figure S5E). Notably, H3R17me2a levels were reduced by PMA treatment and increased by calphostin C treatment (Figure 5B–D; Figure S5F–H). These findings suggest that the PKC‐mediated phosphorylation of KDM4A enables it to demethylate H3R17me2a. Consequently, this phosphorylation led to the upregulation of *Aurkb* and an increase in H3S10ph levels induced by nocodazole (Figure 5E,F). Our findings suggest a compelling model in which KDM4A functions as a demethylase for H3K9me3 and H3R2me2a during interphase, but it demethylates H3R17me2a upon phosphorylation by PKC during mitosis (Figure 5G). Additionally, as previously reported [17, 36], PKC phosphorylates and inhibits CARM1 during mitosis, a finding we confirmed via PKC‐induced band shifts in CARM1 (Figure 5H; Figure S5H).

To exclude the possibility that CARM1 inhibition alone contributes to the reduction in H3R17me2a, we examined the PKC‐KDM4A axis in CARM1‐KO cells. In both CARM1 WT and KO cells, PMA treatment induced KDM4A band shifts, decreased H3R17me2a levels, and increased nocodazole‐induced mitotic arrest (Figure 5I–K; Figure S5I). These results suggest that the PKC‐mediated phosphorylation of CARM1 inhibits its enzymatic activity, preventing H3R17 methylation. In contrast, phosphorylated KDM4A simultaneously demethylates H3R17me2a, thereby maintaining the unmethylated state of H3R17 during mitosis. Furthermore, we propose that PKCα is the kinase responsible for KDM4A phosphorylation. Consistent with our results from the PMA and calphostin C treatments, overexpression of the catalytically active (CA) mutant of PKCα led to KDM4A phosphorylation (Figure S5J), reducing the levels of H3R17me2a, but not H3K9me3 and H3R2me2a (Figure 5H; Figure S5K). In contrast, overexpression of the dominant negative (DN) mutant exhibited the opposite effects (Figure 5H; Figure S5J,K). Moreover, PKCα‐CA increased nocodazole‐induced H3S10ph levels (Figure S5L), further supporting the role of PKCα in this regulatory process.

### 2.6 Phosphorylation of KDM4A at S504 by PKCα Makes It Erase H3R17me2a as Well as H3K9me3

**FIGURE 6:**
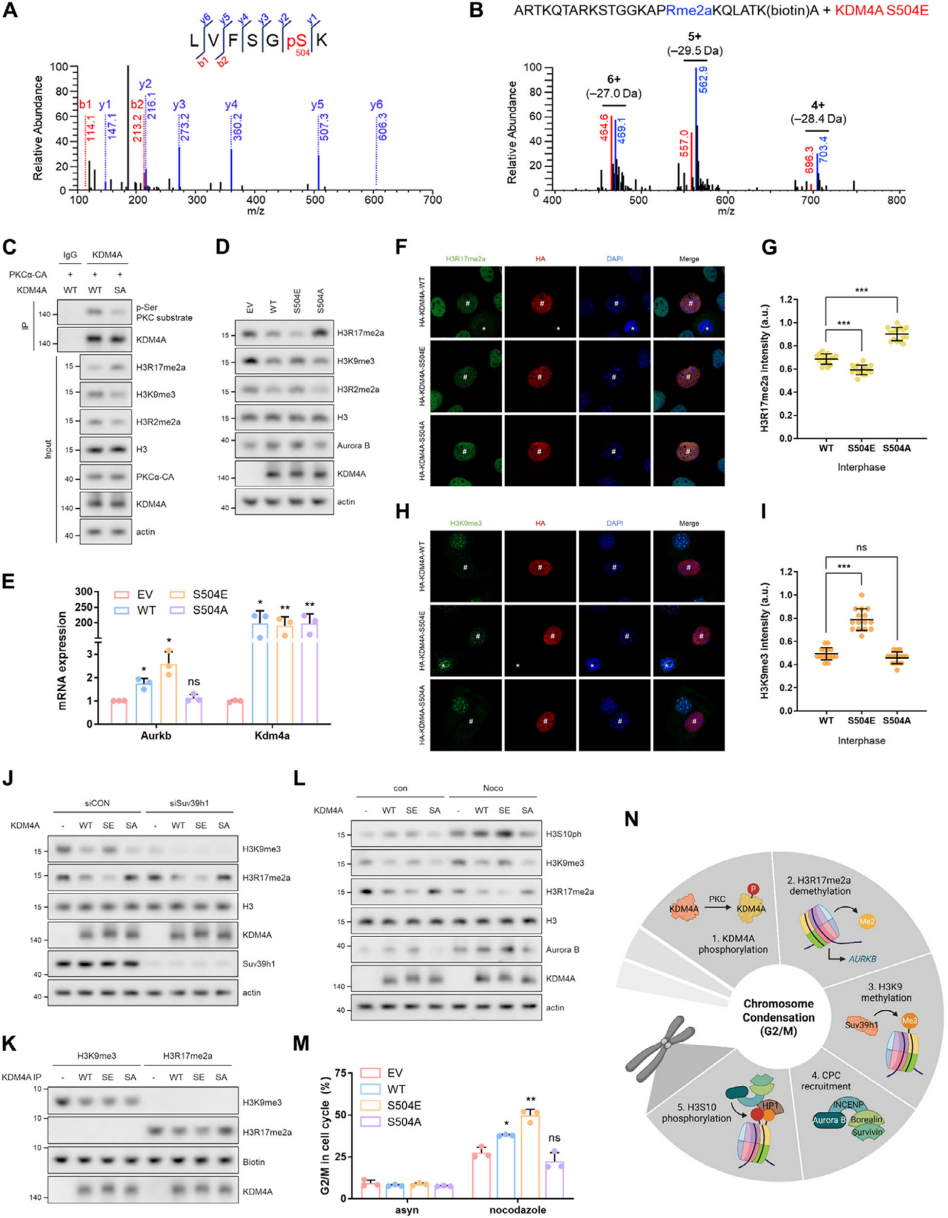
Phosphorylation of KDM4A at S504 by PKCα makes it erase H3R17me2a as well as H3K9me3. (A), Representative MS/MS spectra of peptides containing S504, demonstrating phosphorylation. (B), Mass spectrum obtained by precursor ion scanning of in vitro demethylation assay using bead‐captured KDM4A from cells overexpressing KDM4A‐S504E and biotinylated histone H3 peptides (1–24 aa; ARTKQTARKSTGGKAPR(me2a)KQLATK(biotin)A). Precursor ion peaks derived from methylated (blue) and demethylated (red) peptides are indicated, with charge states and corresponding mass differences annotated. (C), Immunoprecipitation using an anti‐KDM4A antibody in cells co‐transfected with PKCα‐CA and KDM4A (WT or S504A) for 48 h. (D,E), Western blots (D) and mRNA levels (E) in cells overexpressing KDM4A‐WT, ‐S504E, or ‐S504A. (F–I), Representative images and intensities of H3R17me2a (F,G) and H3K9me3 (H,I) were analyzed via immunostaining in cells overexpressing HA‐KDM4A‐WT, ‐S504E, or ‐S504A. (J), Western blots of lysates from cells co‐transfected with Suv39h1 siRNA and KDM4A (WT, S504E, or S504A) plasmid for 48 h. (K), In vitro demethylation assay using beads‐captured KDM4A from cells overexpressing KDM4A‐WT, ‐S504E, or ‐S504A and biotinylated histone H3 peptides (1–24 aa; ARTKQTARK*STGGKAPR*KQLATK(biotin)A). (L,M), Western blots (L) and cell cycle profiles analyzed using FACS (M) in cells treated with nocodazole after transfection with KDM4A‐WT, ‐S504E, or ‐S504A. (N), Schematic representation of the role of H3R17me2a in chromosome condensation.

The Scansite database predicted S504 of KDM4A as a potential PKC phosphorylation site (Figure S6A), which was subsequently confirmed by mass spectrometry analysis (Figure 6A). To investigate whether KDM4A acquires demethylase activity for H3R17me2a through PKCα‐mediated phosphorylation at S504, we generated phosphorylation‐mimetic (S504E) and phosphorylation‐deficient (S504A) mutants of KDM4A. As expected, in vitro demethylation assays using immunoprecipitated KDM4A demonstrated that the S504E mutant, but not WT or S504A, induced a −28 Da mass shift in the H3R17me2a peptide, indicating arginine demethylation (Figure 6B; Figure S6B). Moreover, treatment with PMA or overexpression of the PKCα‐CA mutant induced phosphorylation of KDM4A WT but not the S504A mutant (Figure 6C; Figure S6C,D). PKC activation also failed to decrease H3R17me2a levels in KDM4A S504A‐overexpressing cells, in contrast to KDM4A WT (Figure 6C; Figure S6C,D). Collectively, these results identify S504 as the major PKCα‐dependent phosphorylation site on KDM4A and demonstrate its critical role in conferring H3R17me2a demethylase activity. Consistently, the overexpression of KDM4A S504E reduced H3R17me2a levels, thereby increasing *Aurkb* transcription, whereas the overexpression of S504A had no such effect. The relatively modest activity of KDM4A WT is likely attributable to partial phosphorylation by upstream kinases within the cellular context (Figure 6D,E; Figure S6E). Next, we performed immunostaining to assess how phosphorylation at S504 affects the demethylase activity of KDM4A. The overexpression of S504E significantly reduced H3R17me2a levels in both interphase and mitosis compared to the WT, whereas overexpression of S504A led to an increase in H3R17me2a levels (Figure 6F,G; Figure S6F,G). Conversely, H3K9me3 levels increased with S504E overexpression but not with S504A overexpression (Figure 6H,I; Figure S6H,I). These results confirmed that the phosphorylation of KDM4A at S504 is essential for its H3R17me2a demethylation function.

Next, we investigated how H3K9me3 levels further increased upon S504E overexpression. This increase could either be due to the loss of KDM4A demethylase activity, inhibiting H3K9me3 demethylation, or because reduced H3R17me2a enhances Suv39h1 binding, promoting H3K9 methylation. To clarify these possibilities, we performed Suv39h1 knockdown experiments and in vitro demethylation assays. Suv39h1 knockdown markedly decreased H3K9me3 levels regardless of the S504 mutation (Figure 6J). Moreover, KDM4A WT, S504E, and S504A all efficiently demethylated H3K9me3 in vitro (Figure 6K), demonstrating that KDM4A S504E erases H3R17me2a while retaining H3K9me3 demethylation activity. The reduction in H3R17me2a levels leads to the recruitment of Suv39h1 to chromatin, which subsequently restores H3K9me3 levels. Finally, we confirmed that the phosphorylation of KDM4A at S504 influenced mitotic entry. Overexpression of S504E reduced H3R17me2a levels, increased Aurora B levels, and restored H3K9me3 levels, thereby promoting nocodazole‐induced mitotic arrest. In contrast, the overexpression of S504A had the opposite effect (Figure 6L,M). In conclusion, our study suggests that during mitosis, the PKCα‐induced phosphorylation of KDM4A at S504 not only promotes H3R17me2a demethylation and upregulation of *Aurkb* but also recruits Suv39h1 to chromatin, leading to H3K9me3 restoration and subsequent increase in H3S10ph levels. These sequential events facilitate complete chromosome condensation and mitotic entry (Figure 6N).

## 3 Discussion

This study provides critical insights into the molecular mechanisms that regulate mitosis by elucidating the interplay between active and repressive histone marks during chromosome condensation. Specifically, levels of the active histone mark H3R17me2a decrease during the early stages of mitosis, which are restored at later stages. This is inversely correlated with the repressive mark H3K9me3, and this finely tuned process governs the chromatin recruitment of the CPC and mitotic progression. One of the key contributions of this study to the literature is the elucidation of how H3R17me2a levels decrease. By identifying KDM3A and KDM4A as demethylases for H3R17me2a, we identified novel players in the epigenetic regulation of mitosis. KDM4A demethylates H3K9me3 and H3R2me2a during interphase; however, during mitosis, phosphorylation by PKCα alters its substrate preference, enabling H3R17me2a demethylation and a relative reduction in activity toward H3R2me2a (Figure S6J,K). This phosphorylation‐dependent shift in substrate preference appears to be specific to KDM4A. Notably, KDM3A lacks a serine/threonine residue equivalent to KDM4A S504, and no phosphorylation‐dependent changes in KDM3A were detected under our experimental conditions. Nevertheless, publicly available databases, including PhosphoSitePlus (www.phosphosite.org) and Scansite, predict that KDM3A may be phosphorylated at several residues, such as T308, S446, T668, or Y1303, particularly during the G2/M phase. These observations raise the possibility that KDM3A activity could be regulated by phosphorylation or other PTMs in a context‐dependent manner. Elucidating such regulatory mechanisms may help reconcile the discrepancy between in vitro and in vivo demethylation activities and contribute to a more comprehensive understanding of the biological roles of RDMs. Investigating these possibilities represents an important direction for future studies and is the focus of our ongoing work.

During the G2/M phase, PKCα‐mediated phosphorylation suppresses CARM1‐dependent H3R17 methylation [15, 17, 36] and simultaneously promotes KDM4A‐mediated demethylation of H3R17me2a, resulting in an overall reduction of H3R17me2a levels. These events enhance the chromatin recruitment of Suv39h1 to load H3K9me3, followed by the sequential loading of H3S10ph, thus completing chromosome condensation. Among the many factors involved in chromosome condensation, CPC recruitment to the chromatin plays a pivotal role [10]. In the late G2 phase, HP1 bound to H3K9me3 recruits INCENP to the centromeric heterochromatin, followed by Aurora B‐mediated H3S10ph [8, 9], which displaces HP1 from chromosomes in a process known as the histone H3 methyl‐phospho switch [37, 38]. Early in mitosis, H3R2me2a is mediated by PRMT6 and positions the CPC on the chromosome arms, after which Haspin‐mediated H3T3ph relocates the CPC to the centromere, promoting H3S10ph and facilitating chromosome condensation [11]. In late mitosis, the dephosphorylation of H3T3ph and the mitotic kinesin‐like protein 2 reposition CPC from the centromere to the central spindle, initiating cytokinesis [39, 40]. Although additional histone modifications undergo dynamic changes during mitosis, their functional significance remains unclear.

Our study demonstrates that a reduction in H3R17me2a levels is a critical early event driving chromosome condensation, as it is associated with increased Aurora B expression and enhanced recruitment of Suv39h1 to the chromatin. The molecular mechanism by which H3R17me2a loss leads to elevated Aurora B levels remains unresolved. Given that H3R17me2a is generally associated with transcriptionally active chromatin, its reduction may indirectly influence Aurora B mRNA expression or stability through broader chromatin changes rather than via a direct regulatory interaction. Importantly, our findings define H3R17me2a reduction as an early chromatin transition that links transcription‐associated histone modification dynamics to mitotic chromatin architecture. This highlights the significance of crosstalk among histone modifications in coordinating chromosome condensation and mitotic progression, emphasizing a critical role for histone arginine methylation in mitotic control.

## 4 Experimental Section

### 4.1 Cell Culture

10T1/2, MEF, and HEK293T cells were cultured in Dulbecco's modified Eagle's medium (HyClone, Logan, UT, USA) supplemented with 10% fetal bovine serum (HyClone) and 100 U/mL penicillin/streptomycin (HyClone). All cells were maintained at 37°C in a humidified incubator with 5% CO_2_.

### 4.2 Chemicals, Plasmids, and Antibodies

4′,6‐Diamidino‐2‐phenylindole (DAPI, D1306) was obtained from Thermo Fisher Scientific (Waltham, MA, USA). BI2536 (HY‐50698) and TP‐064 (HY‐114965) were purchased from MedChemExpress (Monmouth Junction, NJ, USA). Calphostin C (C6303), nocodazole (487928), PMA (P8139), and RO‐3306 (SML0569) were purchased from Sigma–Aldrich (St. Louis, MO, USA).

Flag‐KDM (KDM3A, KDM4E, KDM5C, KDM7B, and JMJD6) plasmids were generated by GenScript Biotech Corporation (Piscataway, NJ, USA). The GFP‐CARM1 plasmid was provided by Dr. Mark T. Bedford (University of Texas, MD Anderson Cancer Center). As described previously [41], GFP‐CARM1 (E266Q, enzyme dead mutant) was generated by Muta‐Direct Site‐Directed Mutagenesis Kit (iNtRON Biotechnology, Seongnam, Korea, #15071) according to the manufacturer′s protocol. The HA‐CARM1 (#81118), HA‐KDM4A (#24180), HA‐KDM4C (#24214), HA‐KDM6B (#24167), HA‐PKCα (#21232), HA‐PKCα‐CA (#21234), and HA‐PKCα‐DN (#21235) plasmids were purchased from Addgene (Watertown, MA, USA). The histone H3‐Flag‐HA vector was purchased from OriGene Technologies, Inc (Rockville, MD, USA). As described previously [11], histone H3R2A, H3R2K, and H3S10A mutants were generated using a Muta‐Direct Site‐Directed Mutagenesis Kit (iNtRON Biotechnology). Histone H3R17A, H3R17F, H3R17K, and HA‐CARM1‐ΔNLS mutants were produced by Bionics (Seoul, Korea).

The following antibodies were used for immunoblotting, immunoprecipitation, or immunostaining: actin (Santa Cruz Biotechnology, Dallas, TX, USA, sc‐47778, 1:10000), Aurora B (Abcam, Cambridge, UK, ab2254, 1:5000), Borealin (Novus Biologicals, Centennial, CO, USA, NBP1‐89951, 1:2000), CARM1 (Bethyl Laboratories, Montgomery, TX, USA, A300‐421A, 1:5000), Cyclin B1 (Cell Signaling Technology, Danvers, MA, USA, #12231, 1:2000), Flag (Cell Signaling Technology, #2368 and #8146, 1:5000), HA (Cell Signaling Technology, #3724, 1:5000), histone H3 (Cell Signaling Technology, #9715, 1:5000), H3K9me2 (EpigenTek, Farmingdale, NY, USA, #A‐4035, 1:5000), H3K9me3 (Merck Millipore, Billerica, MA, USA, 07–442, 1:5000 and Diagenode, Denville, NJ, USA, C15200146, 1:500), H3R2me2a (Merck Millipore, 07–585, 1:2000), H3R17me2a (Abcam, ab8284, 1:2000), H3R26me2a (EpigenTek, #A‐3707, 1:2000), H3S10ph (Cell Signaling Technology, #9701, 1:5000), H3T3ph (Abcam, ab78351, 1:5000), INCENP (Thermo Fisher Scientific, 39–2800, 1:2000), KDM1A (Cell Signaling Technology, #2184, 1:5000), KDM3A (Proteintech, Rosemont, IL, USA, 12835‐1‐AP, 1:2000), KDM4A (Proteintech, 29977‐1‐AP, 1:5000), KDM4C (Novus Biologicals, NBP1‐49600, 1:5000), PRMT6 (Bethyl Laboratories, Montgomery, A300‐929A, 1:2000), p‐Ser (Santa Cruz Biotechnology, sc‐81514, 1:1000), p‐Ser PKC substrate (Cell Signaling Technology, #6967, 1:2000), Sp1 (Merck Millipore, CS200631, 1:5000), Survivin (Abcam, ab76424, 1:2000), Suv39h1 (Cell Signaling Technology, #8729, 1:2000 and Santa Cruz Biotechnology, sc‐23961, 1:500), Suv39h2 (Proteintech, 11338‐1‐AP, 1:1000), and α‐tubulin (Santa Cruz Biotechnology, sc‐5286, 1:500). Horseradish peroxidase (HRP)‐conjugated secondary antibodies (111‐035‐003 and 115‐035‐003) were purchased from Jackson ImmunoResearch Laboratories (West Grove, PA, USA). Alexa Fluor‐conjugated secondary antibodies (A90‐116D4, A90‐138D2, A120‐101D4, and A120‐101F) were purchased from Bethyl Laboratories.

### 4.3 Immunostaining and Live Cell Imaging

As described previously [42], cells plated on coverslips were fixed with 4% paraformaldehyde and permeabilized with 0.5% Triton X‐100 for 15 min at room temperature. The cells were incubated with the primary antibodies overnight at 4°C, followed by incubation with the secondary antibodies conjugated to fluorescent. The cells were then visualized using a Zeiss LSM 710 Confocal Microscope (Carl Zeiss, Oberkochen, Germany), and images were analyzed using ZEN or ImageJ/Fiji software.

For time‐lapse microscopy, 10T1/2 cells stably expressing GFP‐H2B were cultured in Leibovitz's L‐15 medium (Thermo Fisher Scientific) supplemented with 10% fetal bovine serum (HyClone) and 2 mm L‐glutamine (Thermo Fisher Scientific). The cells were placed in a sealed growth chamber heated to 37 °C and observed using a Zeiss Axiovert 200 M microscope (Carl Zeiss). Images were acquired every 3 min for 5 h using AxioVision 4.8.2 (Carl Zeiss).

### 4.4 Chromosome Spreading Assay

Chromosome spreading for immunostaining was performed using a modified hypotonic swelling method. Cells grown in 100 mm dishes were treated with nocodazole (150 ng/mL, 2 h) to enrich mitotic cells. Mitotic cells were centrifuged at 1,200 rpm for 3 min at room temperature, and gently resuspended in phosphate‐buffered saline (PBS). Pre‐warmed hypotonic solution (0.8% sodium citrate) was added dropwise while gently tapping the tube, followed by incubation at room temperature for 10 min. Cells were then centrifuged at 1 000 rpm for 10 min, and the pellet was carefully resuspended in 300 µL of the residual solution. The cell suspension was dropped onto coverslips from an approximate height of 10 cm, ensuring that drops did not overlap. Coverslips were immediately fixed with 2.6% paraformaldehyde for 15 min at room temperature, washed three times with PBS, and subjected to blocking and immunostaining according to standard procedures.

### 4.5 Immunoblotting and Immunoprecipitation

As described previously [43], cells were lysed using RIPA buffer (50 mm Tris‐HCl [pH 8], 150 mm NaCl, 0.5% sodium deoxycholate, 0.1% SDS, and 1% Triton X‐100) supplemented with 1× protease and phosphatase inhibitor cocktails (Roche, Basel, Switzerland). The lysates were centrifuged at 16 000 ×*g* for 10 min at 4°C. The protein concentration of the lysate was quantified using the Bradford assay (Bio‐Rad, Hercules, CA, USA) following the manufacturer's instructions.

For immunoprecipitation, the appropriate antibodies were added to the lysates, and the mixtures were incubated overnight at 4°C on a rotator. Thereafter, antibody‐protein complexes were obtained using Protein A/G Sepharose beads (Santa Cruz Biotechnology). After washing twice with a RIPA buffer, the complexes were eluted and analyzed using sodium dodecyl sulfate‐polyacrylamide gel electrophoresis (SDS‐PAGE). Proteins subjected to SDS‐PAGE were transferred to a polyvinylidene fluoride membrane (Merck Millipore) and blocked with 5% skim milk/0.1% Tween 20/Tris‐buffered saline (TBS‐T) for at least 1 h at room temperature. Next, the membranes were incubated with the primary antibodies overnight at 4°C. After washing with TBS‐T, the membranes were incubated with the corresponding HRP‐linked secondary antibodies for 1 h at room temperature. Protein signals were detected using an ECL western blotting substrate (Advansta, Menlo Park, CA, USA).

### 4.6 Histone Extraction

Cells were lysed using RSB buffer (10 mm Tris‐HCl [pH 7.6], 10 mm NaCl, and 3 mm MgCl_2_) and centrifuged at 600 ×*g* for 5 min. The supernatant was discarded, and the pellet was resuspended in an RSB buffer containing 0.5% NP‐40. After incubation, the homogenate was centrifuged at 600 ×*g* for 5 min. The pellet containing nuclei was resuspended in 5 mM MgCl_2,_ and an equal volume of 0.8 M HCl was added. Histones were extracted on ice for 20 min and centrifuged at 17 000 ×*g* for 10 min. After the supernatant was transferred to a new tube, the histones were precipitated with 50% trichloroacetic acid and centrifuged at 12,000 ×*g* for 20 min. The pellet was washed once with acetone‐0.3 M HCl and twice with pure acetone. Finally, the pellets were dried and resuspended in 100 µL of 30 mm Tris‐HCl (pH 8.8).

### 4.7 In Vitro Methylation Assay

After immunoprecipitation with anti‐CARM1 or anti‐PRMT6 antibody, we added beads‐captured proteins to a mixture of H3 peptide and 1 µm S‐adenosyl‐L‐methionine (SAM). After incubation for 1 h at 37°C, the methylation reaction was stopped by adding Laemmli sample buffer to the tube. For lysine methylation, beads‐captured Suv39h1 or recombinant Suv39h1 protein (Sino Biological, Houston, TX, USA) was incubated with total histone extracts and 20 µm SAM in a reaction buffer containing 50 mm Tris‐HCl (pH 8.8), 5 mm MgCl_2_, and 4 mm DTT for 1 h at 37°C.

### 4.8 Cell Synchronization and Flow Cytometry Analysis

To induce G2/M arrest, cells were treated with RO‐3306 (10 µM, 24 h), BI2536 (100 nm, 24 h), or nocodazole (100 ng/mL, 12 h). For metaphase arrest, cells were released in fresh media containing 20 µM MG132 for 2 h after nocodazole treatment.

To analyze the cell cycle profiles, cells were harvested using trypsin and fixed with 70% ethanol for 1 h on ice. The cells were then washed with PBS and incubated with 0.2 mg/mL RNase A (Thermo Fisher Scientific) for 1 h at room temperature. Alexa Fluor 488‐conjugated anti‐H3S10ph antibody was used to distinguish mitotic cells. After harvesting, cells were fixed with 4% formaldehyde for 15 min at room temperature and permeabilized with 90% methanol for 10 min on ice. The cells were washed with PBS and incubated with primary antibody (Alexa Fluor 488‐conjugated H3S10ph, Cell Signaling Technology, #3465, 1:50) for 1 h in the dark. Finally, the cells were stained with 10 µg/mL propidium iodide (Sigma–Aldrich). Data were generated using a FACSCalibur system (BD Biosciences, Franklin Lakes, NJ, USA) and analyzed using FlowJo software.

### 4.9 In Vitro Kinase Assay

Recombinant Aurora B protein (Sino Biological) was incubated with total histone extracts and 10 µm ATP in a reaction buffer containing 25 mm Tris‐HCl (pH 7.5), 5 mm β‐glycerophosphate, 0.1 mm Na_3_VO_4_, 10 mM MgCl_2_, and 1 mm DTT for 1 h at 37°C. The reaction was terminated by adding the Laemmli sample buffer to the tube.

### 4.10 Quantitative Real‐Time PCR

As described previously [44], total RNA was extracted using TRIsure (Bioline, London, UK), and chloroform was added to the cell lysate. The mixture was then vortexed and incubated on ice for 10 min. After centrifugation at 16 000 ×*g* for 15 min at 4°C, the phase containing RNA was transferred to a new tube, and isopropanol was added. After incubation for 10 min at room temperature, the samples were centrifuged at 16 000 ×*g* for 10 min and the pellet was washed with 70% ethanol. The dried RNA pellet was then resuspended in nuclease‐free water. Next, cDNA was synthesized using a SensiFAST cDNA Synthesis Kit (Bioline), and mRNA expression was analyzed using a QuantStudio 3 Real‐Time PCR System (Applied Biosystems, Foster City, CA, USA), SensiFAST SYBR No‐ROX Kit (Bioline), and the ΔΔCT method. The reaction parameters were as follows: cDNA synthesis at 40°C for 60 min, transcriptase inactivation at 95°C for five min, and PCR cycling at 95°C for 10 s, 58°C for 20 s, and 72°C for 20 s (40 cycles).

### 4.11 Subcellular Fractionation

To isolate chromatin, the cells were resuspended in buffer A (10 mm HEPES [pH 7.9], 10 mM KCl, 1.5 mM MgCl_2_, 0.34 m sucrose, 10% glycerol, and 1 mm DTT) supplemented with 1× protease and phosphatase inhibitor cocktails. Then, 0.1% Triton X‐100 was added to the mixture, and the cells were incubated for 5 min on ice. Cytoplasmic proteins were separated from the nuclei via centrifugation at 1,300 ×*g* for 4 min. Nuclei were washed once in buffer A, and then lysed in buffer B (3 mm EDTA, 0.2 mm EGTA, and 1 mm DTT) supplemented with 1× protease and phosphatase inhibitor cocktails for 30 min on ice. Chromatin was then separated from the nucleoplasmic proteins via centrifugation at 1 700 ×*g* for 4 min, washing once with buffer B, and centrifugation at 10 000 ×*g* for 1 min. The final chromatin pellet was resuspended in Laemmli sample buffer.

### 4.12 PTM Analysis

KDM4A immunoprecipitates were resolved on an 8% SDS‐PAGE gel and visualized by Coomassie Brilliant Blue staining. Protein bands were excised and transferred to fresh tubes. Gel pieces were destained by washing twice with 50% ethanol, followed by two washes with distilled water for 10 min each. The gels were then washed with 25 mm ammonium bicarbonate in 50% acetonitrile for 10 min. Reduction was performed using 10 mm dithiothreitol at 56°C for 1 h, followed by alkylation with 55 mm iodoacetamide in the dark at room temperature for 45 min. Proteins were subsequently digested with trypsin. Tryptic peptides were extracted, dried using a low‐temperature SpeedVac, and desalted using C18 ZipTips (Merck Millipore).

Phosphopeptides were analysed using a Q Exactive HF‐X mass spectrometer (Thermo Fisher Scientific) coupled to an Ultimate 3000 UHPLC system. Peptides were loaded onto a trap column and separated on an analytical column (PepMap RSLC, 3 µm, 100 Å, 75 µm × 50 cm). One microgram of each sample was injected and eluted using a linear gradient of 5–30% acetonitrile in 0.1% formic acid at a flow rate of 300 nL min^−^^1^ over 60 min.

For site‐specific identification of KDM4A phosphorylation at S504, peptides were analysed using Parallel Reaction Monitoring (PRM). PRM was performed in positive ion mode under full‐scan conditions with a nanospray voltage of 1.8 kV and a capillary temperature of 275°C. Full MS spectra were acquired over an m/z range of 450–950 at a resolution of 120,000, with a maximum injection time of 100 ms and an AGC target of 3 × 10^6^. Lock mass correction was applied using the polysiloxane ion (m/z 445.12003). Target precursor ions (m/z 409.196, 2+ and 273.133, 3+ for LVFSGpSK) were isolated following full MS scans, with an isolation width of 2.0 m/z. PRM spectra were acquired at a resolution of 30,000 with a maximum injection time of 32 ms, an AGC target of 2 × 10^5^, and a loop count of 8. PRM data were analysed using Mascot (version 2.8.0.1) against a *H. sapiens* KDM4A protein database downloaded from UniProtKB (release April 2025). Search parameters included tryptic digestion with up to two missed cleavages, acetylation (protein N‐terminus), oxidation (Met), and phosphorylation (Ser) as variable modifications. Peptide mass tolerance was set to 5 ppm and fragment mass tolerance to 0.03 Da. Identifications were filtered at a false discovery rate (FDR) of <1% using a decoy database strategy.

### 4.13 In Vitro Demethylation Assay

H3 peptides were incubated with beads‐captured KDM4A protein, α‐ketoglutarate (Sigma–Aldrich; 100 µm), sodium ascorbate (Sigma–Aldrich; 100 µm), and ammonium ferrous sulfate hexahydrate (Sigma–Aldrich; 50 µm) for 1 h at 37°C. The reaction was terminated by adding Laemmli sample buffer to the tube.

For the analysis of demethylation, 1 µL of the reaction mixture was injected into the Vanquish Core HPLC system (Thermo Fisher Scientific) coupled with an ACQUITY UPLC BEH C18 column (100 × 2.1 mm, 1.7 µm) connected with an ACQUITY UPLC BEH C18 VanGuard pre‐column (5 × 2.1 mm, 1.7 µm) (Waters, MA, USA). The mobile phase A was 0.1% formic acid in water, and the mobile phase B was 0.1% formic acid in acetonitrile. The column temperature was set to 65°C. Gradient elution was conducted at a flow rate of 0.4 mL/min as follows: 0–3 min, 3% phase B; 3–10 min, phase B was ramped to 80%; 10–12 min, phase B was ramped to 95%; 12–15 min, 95% phase B; 15–18 min, phase B was ramped down to 3%; 18–25 min, 3% phase B for post‐run. Methylation‐dependent peptide mass differences were analyzed in precursor ion scanning (PIS) mode for the common biotin product ion (m/z 227.0) using a TSQ Vantage triple‐quadrupole mass spectrometer controlled by Xcalibur software (Thermo Fisher Scientific). The collision energy was set to 30 V, with a precursor ion scan range of m/z 400–800 and a scan time of 1 s.

### 4.14 Statistical Analysis

All statistical analyses were performed using Prism software (GraphPad ver 10). Data are representative of independent experiments and are presented as the mean ± standard deviation (*n ≥* 3). Data from two groups were compared using an unpaired *t*‐test for independent samples. Statistical significance was set at *p* < 0.05. ^*^ *p* < 0.05, ^**^ *p* < 0.01, and ^***^ *p* < 0.001.

## Author Contributions

Conceptualization was carried out by Y.C and Y.K.K. methodology was developed by Y.C., J.W.H., G.H.H., S.L., D.G.S., and S.N.K. investigation was performed by Y.C., J.W.H., D.G.S., and S.N.K. visualization was conducted by Y.C; funding acquisition, project administration, and supervision were undertaken by Y.K.K; the original draft was written by Y.C and Y.K.K., and review and editing were performed by Y.C and Y.K.K.

## Conflicts of Interest

The authors declare no conflict of interest.

## Supporting information




**Supporting File 1**: advs74599‐sup‐0001‐SuppMat.docx.

**Supporting File 2**: advs74599‐sup‐0002‐VideoS1.mov.

## Data Availability

All data are available in this article and the Supplementary Information. Additional information is available from the corresponding author upon reasonable request.
