# Supplementary material for: KDM4A Erases the H3R17me2a Mark, Facilitating Chromosome Condensation
Source: Adv Sci (Weinh). 2026 Feb 26;13(26):e14281. doi: 10.1002/advs.202514281 (PMC13159125; doi:10.1002/advs.202514281)
Supplement: Supplementary file 1 — Supporting File 1: advs74599‐sup‐0001‐SuppMat.docx. [file ADVS-13-e14281-s001.docx]

**Supporting Information**

**KDM4A erases the H3R17me2a mark, facilitating chromosome condensation**

Yena Cho^1,2^, Jee Won Hwang^2^, Gyu Hwan Hyun^2^, Sangkyu Lee^3^, Dae-Geun Song^4,5^, Su-Nam Kim^4,5^, and Yong Kee Kim^1,2,*^

^1^Muscle Physiome Research Center and Research Institute of Pharmaceutical Sciences, Sookmyung Women’s University, Seoul 04310, Republic of Korea

^2^College of Pharmacy, Sookmyung Women’s University, Seoul 04310, Republic of Korea

^3^School of Pharmacy, Sungkyunkwan University, Suwon 16419, Republic of Korea

^4^Natural Products Research Institute, KIST Gangneung, Gangneung 25451, Republic of Korea

^5^Natural Product Applied Science, KIST School, University of Science and Technology, Gangneung 25451, Republic of Korea

***Correspondence:**

Yong Kee Kim, Ph.D., E-mail: [yksnbk@sookmyung.ac.kr](mailto:yksnbk@sookmyung.ac.kr), Tel: +82-2-2077-7688, Fax: +82-2-710-9871

**Supplementary figures**

Figure S1 ~ S6.

Supplementary Video 1.


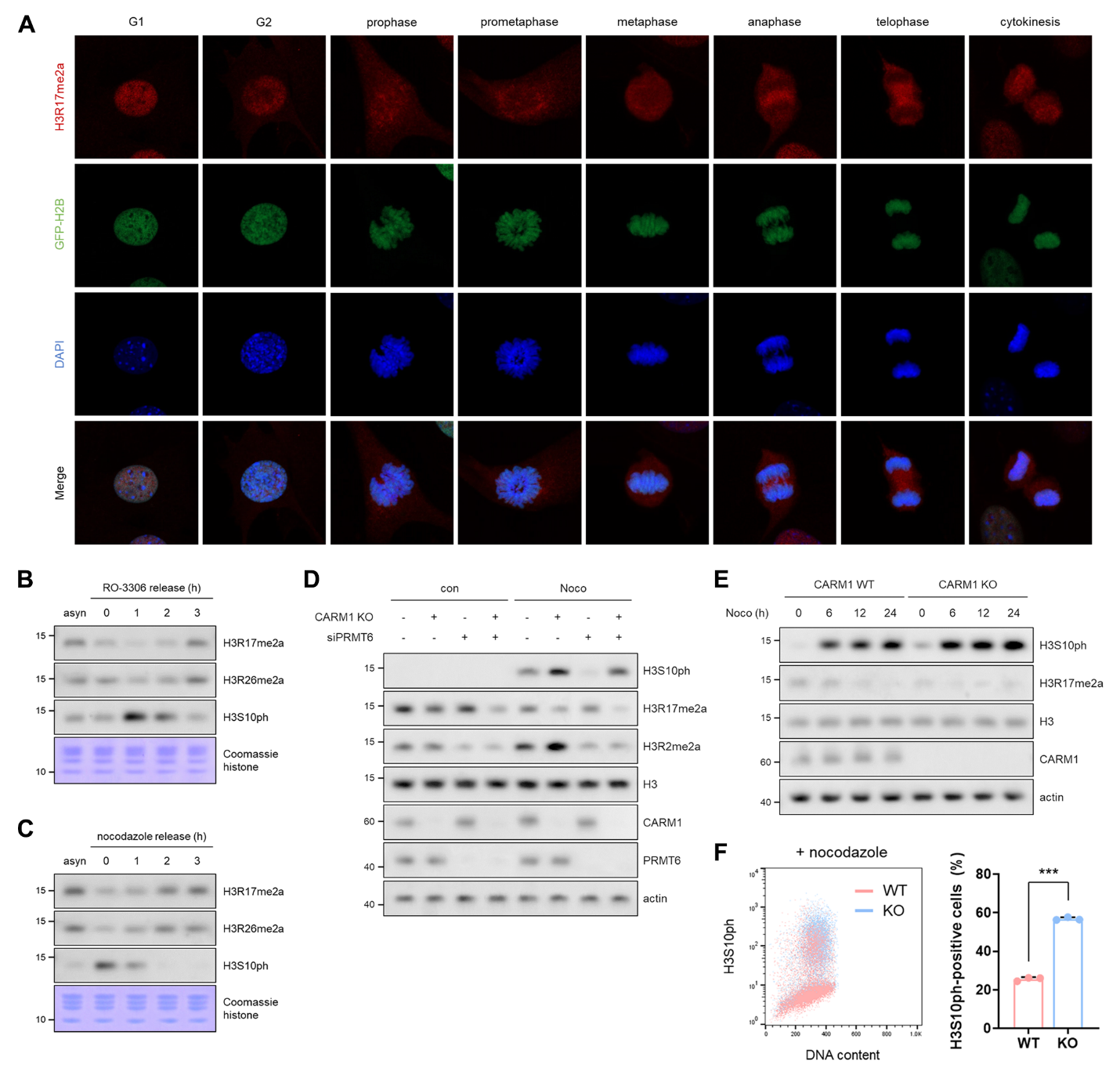
**Figure S1. H3R17me2a reversibly decreases to facilitate chromosome condensation during mitosis**

**A**, Confocal images of H3R17me2a (red), GFP-H2B (green), and DAPI (blue) in 10T1/2 cells stably expressing GFP-H2B throughout the cell cycle. **B**,**C**, Western blots of histones from 10T1/2 cells arrested at the G2 phase (**B**) or prometaphase (**C**) and then released. **D**, Western blots of lysates from CARM1-WT or -KO cells treated with nocodazole after PRMT6 knockdown. **E**, Western blots of lysates from CARM1-WT or -KO cells treated with nocodazole for 6, 12, or 24 h. **F**, Levels of H3S10ph measured by FACS in CARM1-WT or -KO cells. Data are presented as the mean ± SD (n = 3).


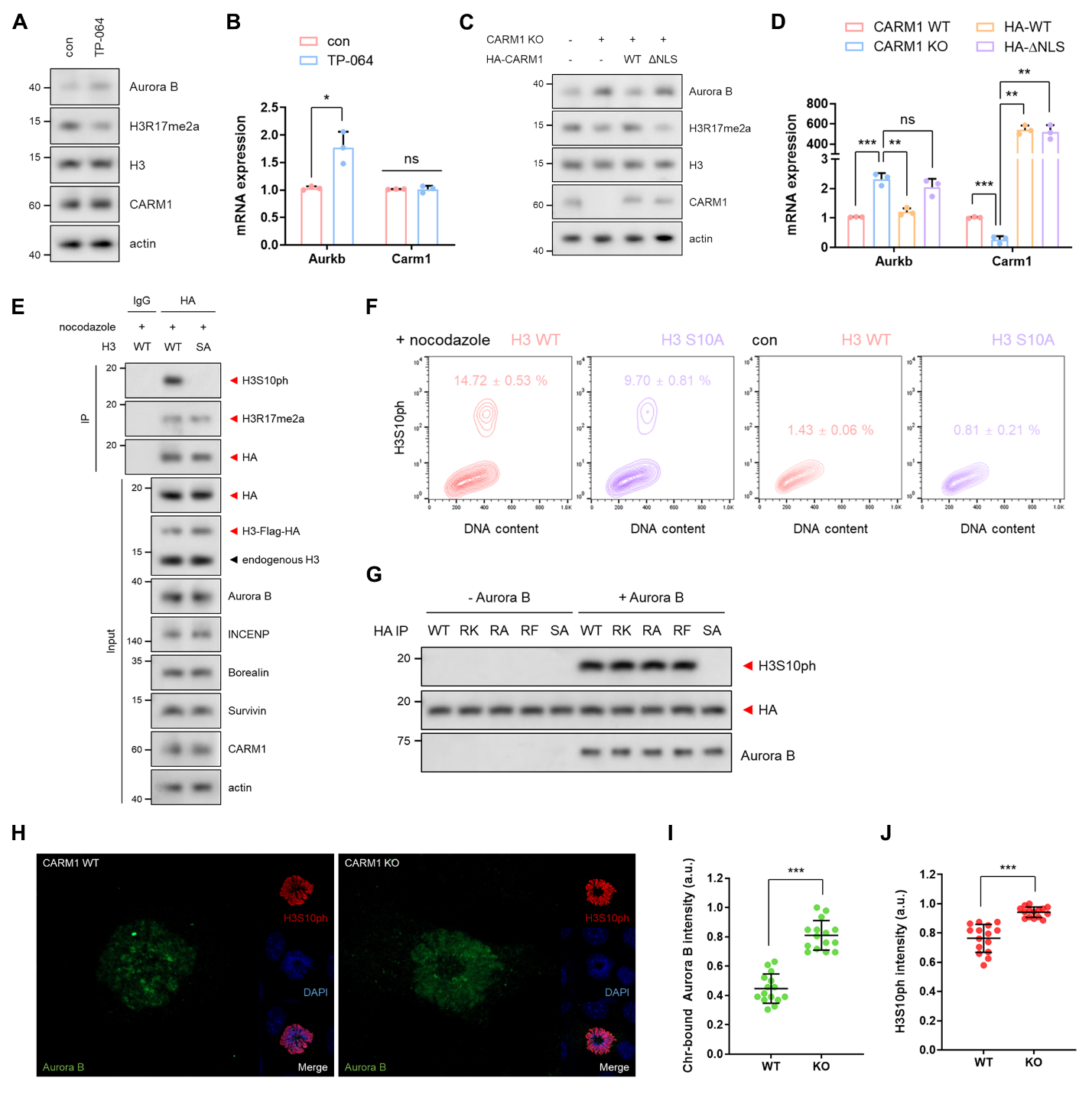


**Figure S2. Reduction in H3R17me2a increases Aurora B levels and chromatin binding, inducing H3S10ph**

**A**, Western blots of lysates from 10T1/2 cells treated with 1 µM TP-064 for 72 h. **B**, mRNA levels of *Aurkb* and *Carm1* in cells treated with a CARM1 inhibitor. **C,D**, Western blots (**C**) and mRNA levels (**D**) in CARM1-WT, and -KO cells transfected with CARM1-WT or -ΔNLS for 48 h. **E**, Immunoprecipitation using an anti-HA antibody in cells overexpressing H3-Flag-HA-WT or -S10A. Red arrowheads indicate Flag-HA-tagged H3, whereas black arrowheads indicate endogenous H3. **F**, Levels of H3S10ph measured by FACS in cells overexpressing H3-Flag-HA-WT or -S10A. Data are presented as the mean ± SD (n = 3). **G**, *In vitro* kinase assay using beads-captured HA from cells overexpressing H3-Flag-HA (WT, R17K, R17A, R17F, or S10A) and recombinant Aurora B protein. Red arrowheads indicate Flag-HA-tagged H3. **H,I,J**, Confocal images of Aurora B (green), H3S10ph (red), and DAPI (blue) in CARM1-WT or -KO cells (**H**). Quantification of chromosome-bound Aurora B (**I**) and H3S10ph intensities (**J**).


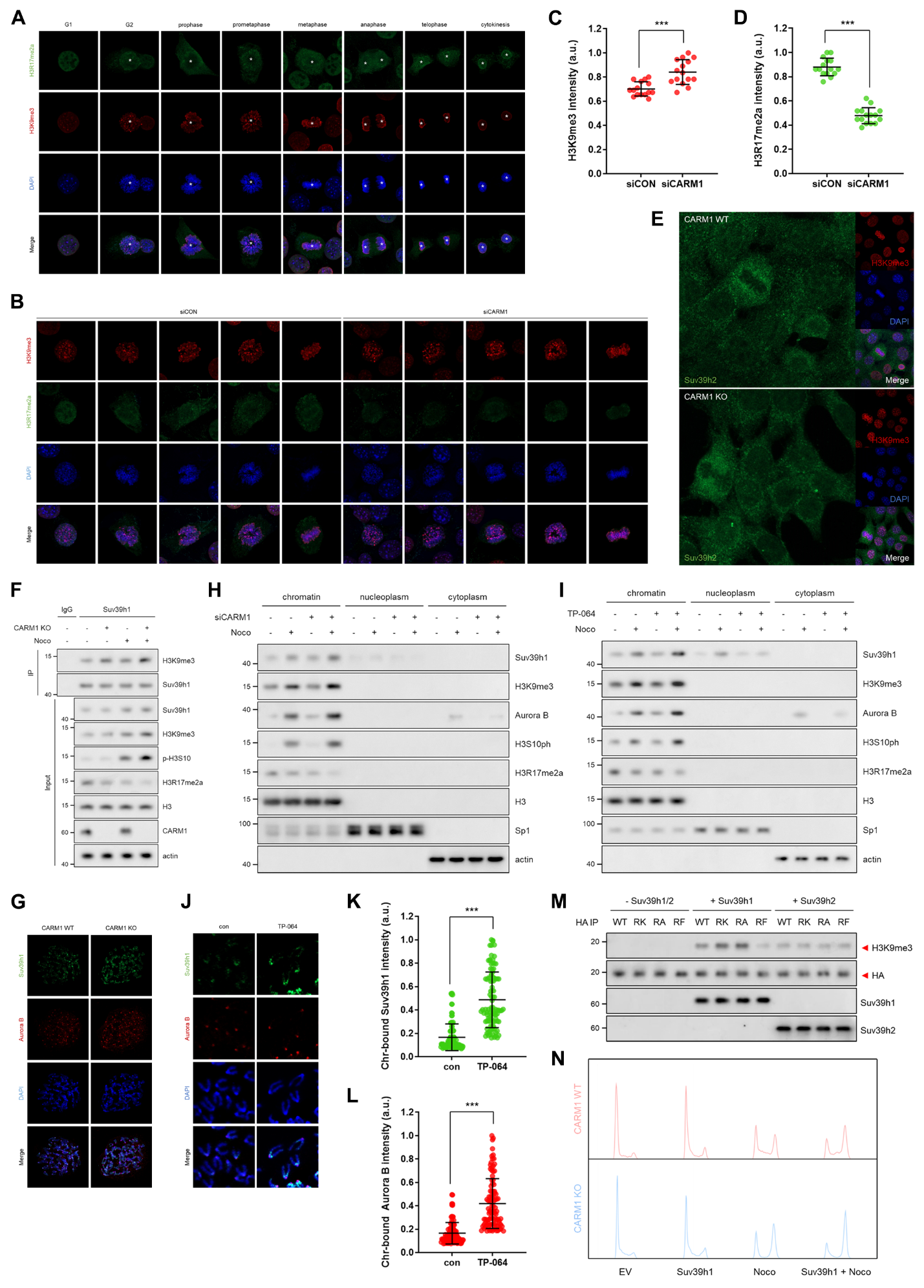


**Figure S3. Reduction in H3R17me2a during mitosis enhances the binding of Suv39h1 to chromatin, leading to an increase in H3K9me3**

**A**, Confocal images of H3R17me2a (green), H3K9me3 (red), and DAPI (blue) in 10T1/2 cells throughout the cell cycle. Representative images corresponding to the quantitative analysis shown in Figure 3E. **B,C,D,** Confocal images of H3K9me3 (red), H3R17me2a (green), and DAPI (blue) in CARM1-depleted mitotic cells (**B**). Quantification of the intensities of H3K9me3 (**C**) and H3R17me2a (**D**). **E**, Confocal images of Suv39h2 (green), H3K9me3 (red), and DAPI (blue) in CARM1-WT or -KO cells. **F**, Immunoprecipitation using an anti-Suv39h1 antibody in CARM1-WT or -KO cells treated with nocodazole. **G**, Confocal images of nocodazole-arrested CARM1-WT or -KO chromosome spreads. Suv39h1 (green), Aurora B (red), and DAPI (blue). **H**,**I**, Western blots of the chromatin fractions from 10T1/2 cells treated with nocodazole after the knockdown (**H**) or inhibition (**I**) of CARM1. **J,K,L,** Confocal images of Suv39h1 (green), Aurora B (red), and DAPI (blue) in nocodazole-arrested 10T1/2 chromosome spreads. Cells were treated with TP-064 (1 µM) for 72 h (**J**). Quantification of chromosome-bound Suv39h1 (**K**) and Aurora B (**L**) intensities. **M**, *In vitro* methylation assay using beads-captured HA from cells overexpressing H3-Flag-HA (WT, R17K, R17A, or R17F) and recombinant Suv39h1/2 protein. Red arrowheads indicate Flag-HA-tagged H3. **N**, Histograms of the cell cycle analyzed using FACS in CARM1-WT or -KO cells under the indicated conditions.

**
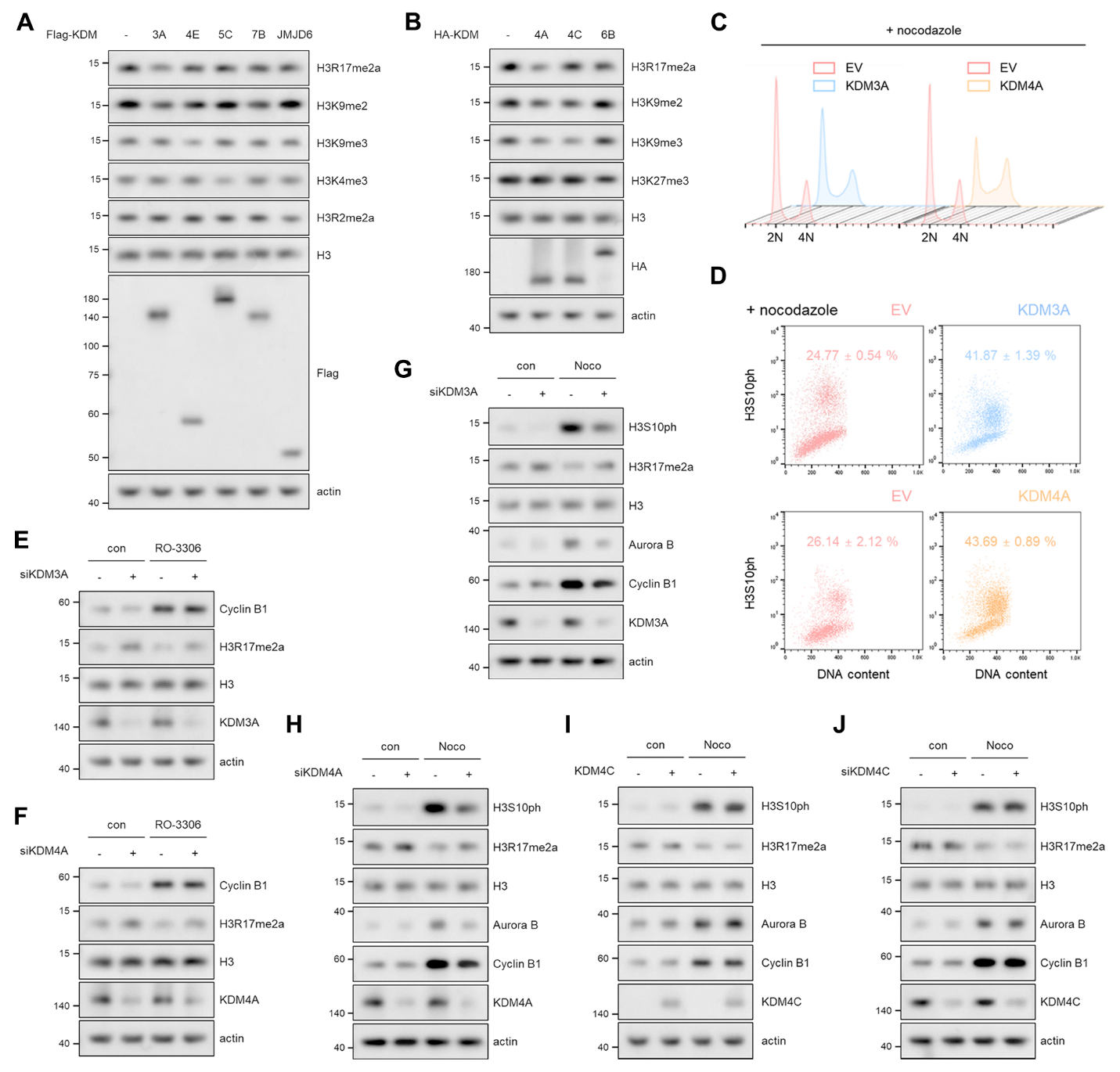
**

**Figure S4. KDM3A and KDM4A possess RDM activity that erases the H3R17me2a mark *in vivo***

**A**,**B**, Western blots of lysates from 10T1/2 cells transfected with KDMs for 48 h. KDM3A, KDM4A, KDM4E, KDM5C, KDM6B, and JMJD6 were reported as potential RDMs. **C**,**D**, Histograms of cell cycle (**C**) and levels of H3S10ph (**D**) analyzed using FACS in KDM3A or KDM4A overexpressing cells. **E**,**F**, Western blots of lysates from cells treated with RO-3306 after the knockdown of KDM3A (**E**) or KDM4A (**F**). **G**,**H**, Western blots of lysates from cells treated with nocodazole after the knockdown of KDM3A (**G**) or KDM4A (**H**). **I**,**J**, Western blots of lysates from cells treated with nocodazole after the overexpression (**I**) or knockdown (**J**) of KDM4C.


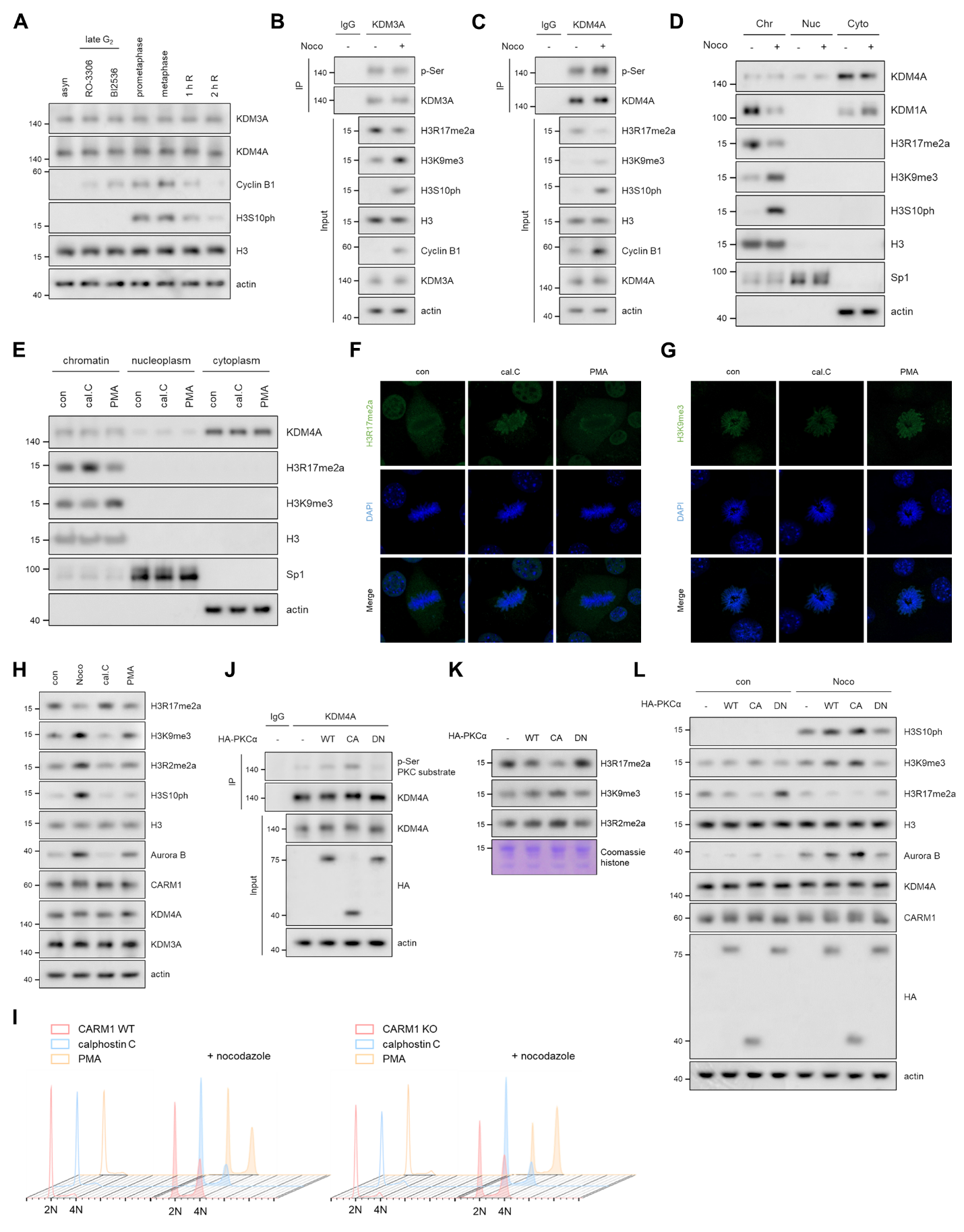


**Figure S5. KDM4A is phosphorylated by PKCα during mitosis, acquiring H3R17me2a demethylation activity**

**A**, Western blots of lysates from 10T1/2 cells arrested in the late G2 phase (RO-3306 or BI2536), prometaphase (nocodazole), or metaphase (nocodazole release with MG132). **B**,**C**, Immunoprecipitation using an anti-KDM3A (**B**) or anti-KDM4A (**C**) antibody in nocodazole-treated cells. **D**,**E**, Western blots of chromatin fractions from cells treated with nocodazole (**D**) or a PKC activity regulator (**E**). **F**,**G**, Confocal images of H3R17me2a (**F**) or H3K9me3 (**G**) (green) and DAPI (blue) in cells treated with calphostin C or PMA for 12 h. **H**, Western blots of lysates from cells treated with nocodazole, calphostin C, or PMA for 12 h. **I**, Histograms of the cell cycle analyzed using FACS in CARM1-WT or -KO cells added with nocodazole after calphostin C or PMA pretreatment. **J**,**K**, Immunoprecipitation using an anti-KDM4A antibody (**J**) and western blots of histones (**K**) from cells overexpressing PKCα-WT, -CA, or -DN. **L**, Western blots of lysates from cells treated with nocodazole after transfection with PKCα-WT, -CA, or -DN.


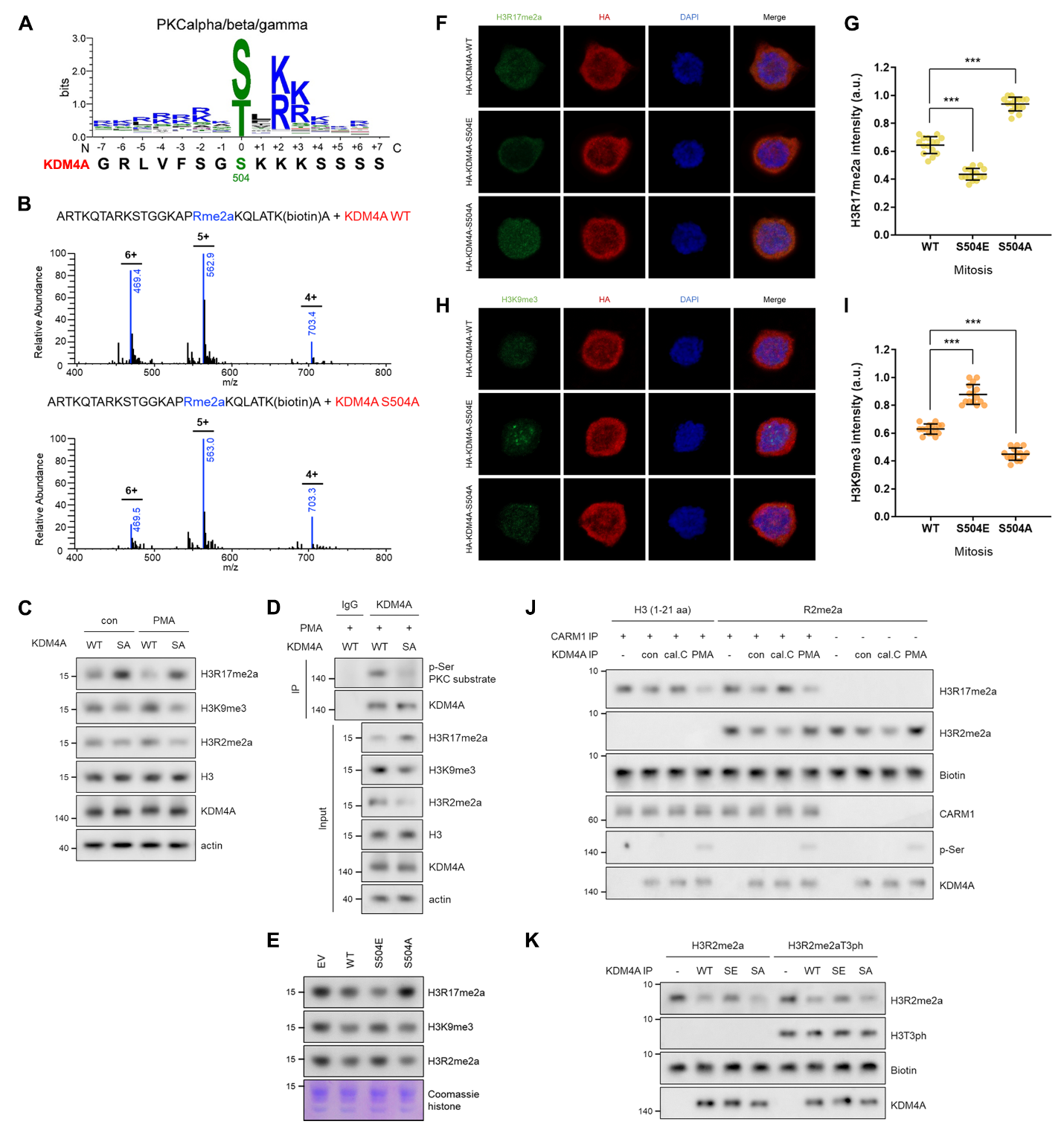


**Figure S6. Phosphorylation of KDM4A at S504 by PKCα makes it erase H3R17me2a as well as H3K9me3**

**A**, Sequence logos of PKC (alpha/beta/gamma) substrates generated from the Scansite database and WebLogo. **B**, Mass spectrum obtained by precursor ion scanning of *in vitro* demethylation assay using bead-captured KDM4A from cells overexpressing KDM4A-WT (top) or -S504A (bottom) and biotinylated histone H3 peptides (1-24 aa; ARTKQTARKSTGGKAPR(me2a)KQLATK(biotin)A). Precursor ion peaks derived from methylated (blue) peptides are indicated, with charge states and corresponding mass differences annotated. No peaks corresponding to demethylated peptides were detected. **C,D,** Western blots of lysates (**C**) and immunoprecipitation using an anti-KDM4A antibody (**D**) from 10T1/2 cells treated with PMA after transfection with KDM4A-WT or -S504A. **E**, Western blots of histones from cells transfected with KDM4A-WT, -S504E, or -S504A for 48 h. **F,G,H,I,** Representative images and intensities of H3R17me2a (**F**,**G**) and H3K9me3 (**H,I**) analyzed *via* immunostaining in mitotic cells overexpressing HA-KDM4A-WT, -S504E, or -S504A. **J**, *In vitro* demethylation assay using beads-captured KDM4A from cells treated with a PKC activity regulator and biotinylated histone H3 peptides (1–21 aa; AR*TKQTARKSTGGKAPRKQLA-GGK(biotin)). **K**, *In vitro* demethylation assay using beads-captured KDM4A from cells overexpressing KDM4A-WT, -S504E, or -S504A and biotinylated histone H3 peptides (1–21 aa; AR*T*KQTARKSTGGKAPRKQLA-GGK(biotin)).

**Supplementary Video 1. Representative time-lapse video of the mitotic division of 10T1/2 cells stably expressing GFP-H2B.** Left: control. Right: TP-064.
